# Persistent Genomic Erosion in Whooping Cranes Despite Demographic Recovery

**DOI:** 10.1111/mec.70088

**Published:** 2025-08-26

**Authors:** Claudia Fontsere, Samuel A. Speak, Andrew J. Caven, Juan Antonio Rodríguez, Xuejing Wang, Carolina Pacheco, Molly Cassatt‐Johnstone, Georgette Femerling, Brigid Maloney, Jennifer Balacco, Joanna Collins, Ying Sims, Linelle Abueg, Olivier Fedrigo, Erich D. Jarvis, Barry K. Hartup, Beth Shapiro, M. Thomas P. Gilbert, Cock van Oosterhout, Hernán E. Morales

**Affiliations:** ^1^ Globe Institute, University of Copenhagen Copenhagen Denmark; ^2^ School of Environmental Sciences University of East Anglia Norwich UK; ^3^ Natural History Museum London UK; ^4^ North of England Zoological Society, Chester Zoo Chester UK; ^5^ International Crane Foundation Baraboo Wisconsin USA; ^6^ Crane Trust Wood River Nebraska USA; ^7^ Biology Department Lund University Lund Sweden; ^8^ Department of Ecology and Evolutionary Biology University of California Santa Cruz Santa Cruz California USA; ^9^ Department of Human Genetics McGill University Montreal Quebec Canada; ^10^ Vertebrate Genome Lab The Rockefeller University New York New York USA; ^11^ School of Veterinary Medicine University of Wisconsin‐Madison Madison Wisconsin USA; ^12^ University Museum, NTNU Trondheim Norway

**Keywords:** captive breeding, conservation genetics, genomic erosion, population genetics—empirical, whooping crane

## Abstract

Integrating in‐situ (wild) and ex‐situ (captive) conservation efforts can mitigate genetic diversity loss and help prevent extinction of endangered wild populations. The whooping crane ( *Grus americana* ) experienced severe population declines in the 18th century, culminating in a collapse to ~20 individuals by 1944. Legal protections and conservation actions have since increased the census population from a stock of 16 individuals to approximately 840 individuals, yet the impact on genomic diversity remains unclear. We analysed the temporal dynamics of genomic erosion by sequencing a high‐quality reference genome, and re‐sequencing 16 historical (years 1867–1893) and 37 modern (2007–2020) genomes, including wild individuals and four generations of captive‐bred individuals. Genomic demographic reconstructions reveal a steady decline, accelerating over the past 300 years with the European settlement of North America. Temporal genomic analyses show that despite demographic recovery, the species has lost 70% of its historical genetic diversity and has increased its inbreeding. Although the modern population bottleneck reduced the ancestral genetic load, modern populations possess more realised load than masked load, possibly resulting in a chronic loss of fitness. Integrating pedigree and genomic data, we underscore the role of breeding management in reducing recent inbreeding. Yet ongoing heterozygosity loss, load accumulation, and persistent effects of historical inbreeding (i.e., background inbreeding) argue against the species' downlisting from its current Endangered status on the IUCN Red List and the Endangered Species Act. The presence of private genetic variation in wild and captive populations suggests that wild‐captive crosses could enhance genetic diversity and reduce the realised load. Our findings emphasise the role of genomics in informing conservation management and policy.

## 1 Introduction

The ongoing sixth mass extinction is reducing population sizes and driving species extinctions worldwide (Barnosky et al. 2011). Conservation strategies, including in‐situ (wild) and ex‐situ (captive) approaches, aim to restore populations and maintain genetic diversity (Braverman 2014). The One Plan Approach integrates these strategies, fostering collaboration among conservation stakeholders to manage both wild and captive populations as a single conservation unit (Sauve et al. 2022). Combining captive breeding with genomic insights is crucial for mitigating threats (Conde et al. 2011; Farquharson et al. 2021; McGowan et al. 2017), but many species have already lost substantial genetic diversity when these efforts begin (e.g., Dussex et al. 2021; Femerling et al. 2023; Feng et al. 2019; Jackson et al. 2022; Sánchez‐Barreiro et al. 2021). Genomic erosion—the combined loss of genetic diversity, accumulation of deleterious mutations (genomic load), and loss of local adaptation—further amplifies the effects of drift and inbreeding, driving populations toward an Extinction Vortex (Bosse and van Loon 2022; van Oosterhout et al. 2022; Fagan and Holmes 2006; Höglund 2009).

Loss of genetic diversity and increasing realised load—the expressed fitness reduction of deleterious alleles—significantly challenge long‐term conservation efforts (Cavill et al. 2024; Klimova et al. 2022; Lacy 1997; van Oosterhout et al. 2022). Genomic erosion unfolds over generations as population decline creates a ‘drift debt’ where reduced effective population sizes (*N*_e_) lead to a delayed loss of genetic diversity and an accumulation of inbreeding and deleterious variants that persist across generations (Gargiulo et al. 2024; Pinto et al. 2024; Liu et al. 2025). This lag can cause traditional conservation assessments, like the IUCN Red List, to underestimate genetic risks highlighted by genomics (van Oosterhout 2024).

While genomic assessments could improve management strategies, comprehensive genetic diversity assessments of pre‐bottleneck populations are rare (Cavedon et al. 2023; Speak et al. 2024). Available assessments are often retrospective, relying on pedigree data or limited genetic markers (e.g., Jackson et al. 2022; Russello and Jensen 2018; Witzenberger and Hochkirch 2011), which reduces accuracy if pedigrees are incomplete or markers unrepresentative of the whole genome (Allendorf et al. 2010). Whole‐genome assessments are essential to understanding inbreeding and genetic load, as bottlenecks elevate realised load by increasing recessive deleterious variants, causing inbreeding depression and fitness loss (Dussex et al. 2023; Bertorelle et al. 2022). In some cases, purifying selection can purge highly deleterious variants, preserving fitness (Bertorelle et al. 2022; Dussex et al. 2023; Robinson et al. 2023). However, bottlenecks often lead to the accumulation of mildly deleterious variants, reducing post‐bottleneck fitness (Dussex et al. 2023). Additionally, the benign environment and equalised genetic contribution in captive breeding can reduce the efficacy of natural selection, allowing deleterious variants to persist (Lynch and O'Hely 2001; Wright et al. 2021).

The whooping crane ( *Grus americana* ) is a powerful case study for evaluating how conservation strategies can mitigate genetic diversity loss in wild and captive populations. The whooping crane is an iconic, endangered North American bird species, recognised by its distinctive white plumage, tall stature, bugling call, and intricate courtship dance. The wild population once likely exceeded 10,000 individuals based on former range estimates, but unregulated hunting, habitat loss, and human disturbance reduced their numbers to around 1300 by 1870 (Allen 1952; Canadian Wildlife Service and U.S. Fish and Wildlife Service 2007). In 1944, the species reached its lowest point of just 21 individuals across two populations. By the 1950s, the population that wintered along the Texas Gulf Coast and migrated through the Central Flyway of North America remained (Canadian Wildlife Service and U.S. Fish and Wildlife Service 2007). Its remote breeding grounds would finally be discovered in Wood Buffalo National Park in northern Canada in 1954 (Allen 1956).

The whooping crane first gained basic protections under the *U.S. Migratory Bird Treaty Act* of 1918 and later more comprehensive protections under the *Endangered Species Preservation Act* of 1966 (U.S. Fish and Wildlife Service [USFWS] 1967). It was officially classified as ‘endangered’ in the first revision of such act in 1969 (USFWS 1970). This designation was affirmed in the Endangered Species Act of 1973, which continues to be the flagship law protecting endangered species in the United States (USFWS 1973). In Canada, the whooping crane was classified as ‘endangered’ by the Committee on the Status of Endangered Wildlife in Canada (COSEWIC) in 1978. The status has been reaffirmed since (Canadian Wildlife Service [CWS] 2011; Canadian Wildlife Service and U.S. Fish and Wildlife Service 2007). Internationally, the species has been listed as Endangered in the IUCN Red List since 1994. A captive breeding program began in 1966, and today the captive population includes over 140 individuals across 18 institutions, aiming to retain over 90% of the remaining species' genetic diversity for the next century through strategic pairings and transfers between institutions (Boardman et al. 2021). Captive breeding minimises mean kinship and inbreeding, with genetically valuable chicks retained to preserve diversity (Boardman et al. 2021; McAbee and Conkin 2025). Captive breeding has supported four reintroduction efforts; two have failed and two are ongoing; the Eastern Migratory Population (EMP, est. 2001) and the Louisiana Non‐migratory Population (LNMP, est. 2011) (Szyszkoski et al. 2025; Louisiana Department of Wildlife and Fisheries [LDWF] 2022; Thompson et al. 2022). Although reintroduced populations show some natural recruitment, they are not yet demographically self‐sustaining and regular releases of captive‐reared individuals are used to reinforce and maintain the size of these populations (Szyszkoski et al. 2025; Caven et al. 2023; LDWF 2022; Thompson et al. 2022). The only remnant migratory wild population, the Aransas‐Wood Buffalo Population (AWBP), has naturally grown from 16 individuals in 1941 to around 540 today in protected wintering and breeding grounds without captive supplementation and it is demographically self‐sustaining (Caven et al. 2023; McAbee and Conkin 2025). Currently, there are over 830 whooping cranes globally across remnant, reintroduced, and captive populations (McAbee and Conkin 2025).

Amid progress toward demographic recovery, the USFWS internally discussed downlisting the whooping crane under the ESA, as information was obtained via a Freedom of Information Act request by the Center for Biological Diversity (Caven et al. 2023; Kurose 2022). Meanwhile, the IUCN aims to reassess the status of all bird species by 2030 to monitor biodiversity progress and guide conservation efforts (IUCN 2019). The whooping crane's status under the ESA and IUCN frameworks depends on demographic, range, and threat factors, which should include genetic health (BirdLife International 2020; Canadian Wildlife Service and U.S. Fish and Wildlife Service 2007; Caven et al. 2023). Genetic and genomic assessments of extant whooping crane populations have been limited, and population viability analyses are hindered by the lack of recent genomic data (Caven et al. 2023; Miller 2024; Miller et al. 2021; Traylor‐Holzer 2019). Moreover, insights from conservation genomic studies are inadequately incorporated in extinction risk assessments (van Oosterhout 2024). Existing research indicates that whooping cranes have lost at least two‐thirds of their pre‐bottleneck mitochondrial DNA haplotypes (Glenn et al. 1999; Jarvi et al. 2001). Captive populations also continue to lose genetic diversity at a modest rate, despite population management best practices (Boardman et al. 2021), which is a likely reflection of the drift debt (Pinto et al. 2024; Jackson et al. 2022).

In this study, we evaluate genomic erosion in the whooping crane using 53 whole genomes spanning the past two centuries across historical, captive, and wild populations. Leveraging a new chromosome‐level assembly, we reconstructed demographic history over the last million years and analysed genetic diversity, inbreeding, and genetic load dynamics for the past 200 years. By estimating both masked load and realised load, we assess the species' vulnerability to future inbreeding depression. Finally, using the complete pedigree for whooping cranes, we evaluate the impact of captive breeding management on mitigating genomic erosion. With this unprecedented genomic insight, we caution against downlisting the species and advocate for an integrated in‐situ (wild) and ex‐situ (captive) management, as proposed in the One Plan approach to conservation.

## 2 Materials and Methods

### 2.1 De Novo Reference Genome Sequence and Assembly

We generated a high‐quality reference genome using the Vertebrate Genome Project's trio v1.6 protocol (Rhie et al. 2021), from two parents and a male offspring collected at the International Crane Foundation (ICF) in 2019. For each parent, we generated Illumina PE 150 bp (88×) and used them to sort the haplotypes of Pacbio continuous long reads (CLR; 233×); the sorted reads were used to generate contigs and then scaffold each haplotype into chromosomes with 10× Genomics (111×), Bionano (417×), Arrima HiC (94×) data. We polished the final maternal and paternal assemblies with the 10× short reads for correcting base call errors and manually curated for structural errors (Figure S1). A detailed summary of insert sizes, library preparation kits, sequencing technology, and list of software can be found in Table S1. We chose the maternal haplotype assembly as the reference, as it was the more complete assembly. The offspring's maternal haplotype assembly has a total length of 1.26 Gb in 39 autosomes, the Z sex chromosome, 929 scaffolds, and the complete mitochondrial genome (Table S1). The assembly has a BUSCO (Simão et al. 2015) completeness of 98.4% (Figure S2). The assembly was fully phased, and both haplotypes are deposited in NCBI (accession numbers: maternal haplotype: GCA_028858705.1 and paternal haplotype GCA_028858595.1).

### 2.2 Sample Collection, DNA Extraction, Library Preparation and Sequencing

#### 2.2.1 Modern Samples

**FIGURE 1:**
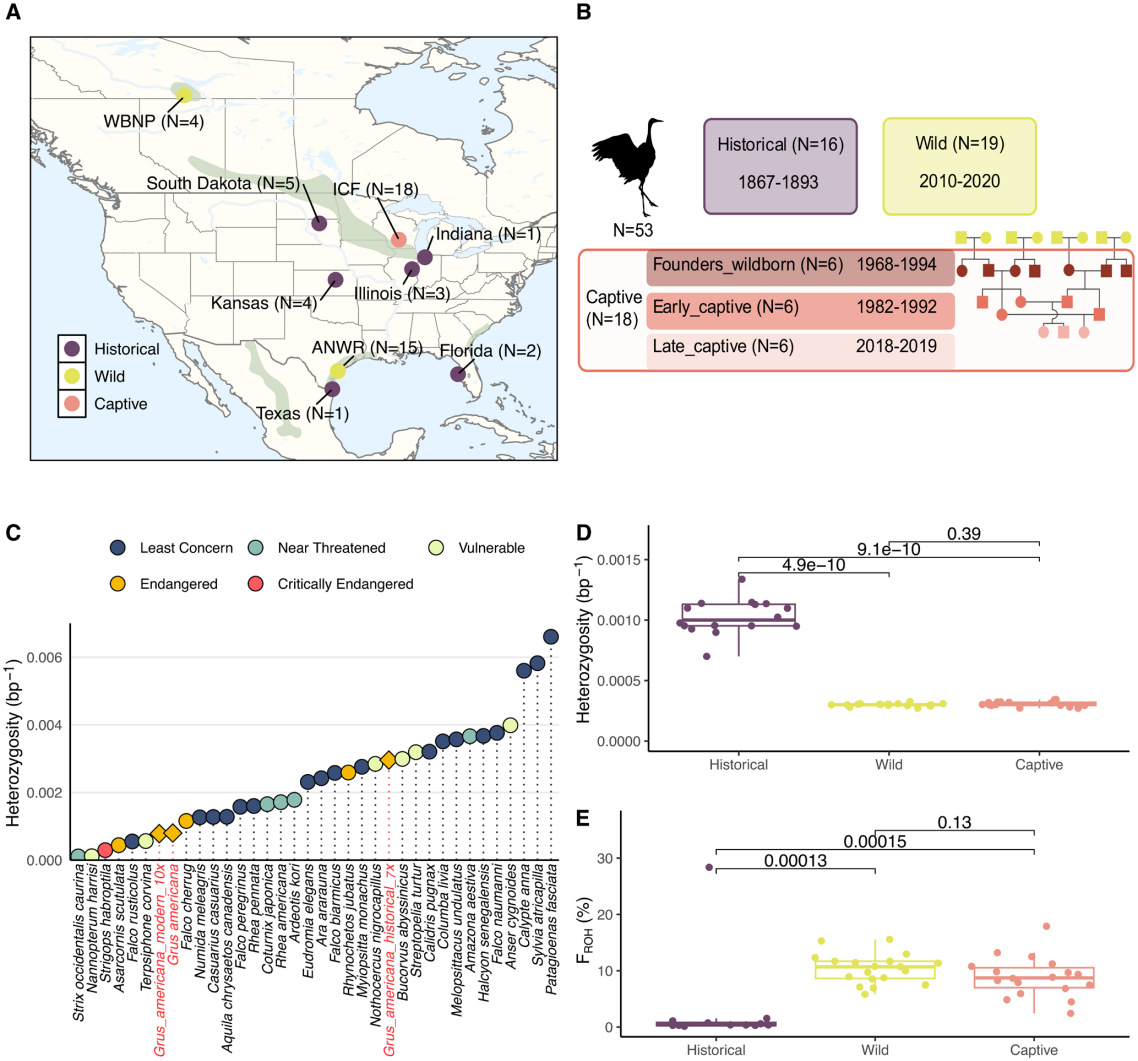
Temporal reduction of genome‐wide heterozygosity and increase of inbreeding in whooping cranes. (A) Map of current distribution of whooping cranes (in green) with the sampling locations of historical, and modern captive (ICF) and wild (WBNP and ANWR, same migratory population) individuals. (B) Experimental design of historical and modern sampling. Captive individuals include three generations: Founders_wildborn are those whose parental generation were wild individuals, and the eggs were hatched in captivity; Early_captive are the first (or second) generation descendants of Founders_wildborn; and Late_captive are captive‐born birds born after at least three generations in captivity. The pedigree shown is an illustrative cartoon of the different generations in captivity, not based on real pedigree data. (C) Genome‐wide heterozygosity (all sites) of the whooping crane (red font; *Grus americana* ) in comparison with other bird species with different IUCN Red List status. Grus_americana_modern_10× represents the average heterozygosity of all modern samples sequenced in this study after downsampling to 10× coverage; *Grus americana* is the reference genome individual at 66.54× coverage; and *Grus_americana_historical_7*× is a historical sample with one of the highest coverages (MCZ‐220028 at coverage 6.99×). Heterozygosity estimates of this historical sample have been corrected with a transition/transversion ratio of 2.89. (D) Statistically significant difference in genome‐wide heterozygosity (only transversions) between historical, wild and captive whooping cranes (Wilcoxon rank‐sum test, Historical vs. Wild *p* value = 4.9e‐10 and Historical vs. Captive *p* value 9.1e‐10), with a 70% loss in heterozygosity since 1893. (E) Statistically significant increase of inbreeding (*F*_ROH_) between historical, wild and captive whooping cranes (Wilcoxon rank‐sum test, Historical vs. Wild *p* value = 0.00013 and Historical vs. Captive *p* value 0.00015), including only samples with coverage > 4×.

We collected 37 modern samples (2007–2020): 18 from the International Crane Foundation (ICF) (2007–2019) and 19 from wild individuals in the Aransas‐Wood Buffalo population, AWBP (Aransas NWR, TX, USA; Wood Buffalo National Park [WBNP], NWT, Canada; 2009–2020). Thus, ANWR and WBNP belong to the single migratory wild population AWBP (Figure 1A; Table S2). ICF, with the largest captive population (36 individuals), maintains detailed pedigree and fitness data for all birds (Liu 2018; McAbee and Conkin 2025). Using this information, we classified each captive individual into three different categories depending on how many generations they have been in the captive programme (Founders_wildborn, *N* = 6; Captive_early, *N* = 6; and Captive_late, *N* = 6). Founders_wildborn are birds of wild origin which founded the captive breeding population (eggs were collected from wild individuals of the AWBP population); Early_captive are captive‐born first‐ or second‐generation descendants of the founders; and Late_captive are captive‐born birds after at least three generations in captivity (Figure 1B).

We extracted DNA from blood stored on FTA cards using the DNEasy Blood and Tissue kit (cat #69506) as described in the protocol for nucleated blood. Briefly, we punched two 2 mm holes from the blood spot on each FTA card using a hole punch. We cleaned the hole punch between samples with 2% NaClO and then with 70% EtOH. We added the punched‐out blood spots to a 1.5 mL tube per sample with 20 μL proteinase‐K and buffer PBS, bringing the final volume to 220 μL. Finally, we performed the DNA isolation as described in the protocol. We converted the modern extractions into genomic libraries using the NEB Ultra II FS kit (cat #E7805L) with enzymatic shearing. We used 100 ng of DNA as input, shearing extracts for 14 min at 37°C, and followed the protocol as described. We size‐selected for final library products of 270–370 bp and amplified the libraries for five cycles. We pooled the libraries at an equimolar ratio and sequenced the genomic libraries on one lane of 2 × 150 S4 Illumina NovaSeq 6000 run at Duke University. Modern genomes were sequenced to an average depth of coverage of 13.4× (ranging from 8.1× to 18.3×) (Table S2).

#### 2.2.2 Historical Samples

We obtained 16 historical toe‐pad samples from Kansas University and Harvard Museum of Comparative Zoology collections (Figure 1; Table S2), dated from 1867 to 1893, representing the pre‐20th‐century bottleneck population. We conducted all historic extractions and genomic library preparations in a dedicated clean lab facility at the UCSC Paleogenomics Lab, following protocols described in Fulton and Shapiro (2019). We used sterile scalpels to subsample approximately 2–3 mm^2^ of tissue per extraction, and mechanically homogenised the tissue in 1.5 mL tubes using 1 mL of digestion buffer optimised for hair and tissue (Gilbert et al. 2007). We isolated DNA following (Dabney et al. 2013) and prepared single‐stranded Illumina compatible genomic libraries following (Kapp et al. 2021). We amplified the libraries as informed by qPCR results, and sequenced the libraries on an in‐house Illumina NextSeq 550 2 × 75 run to assess sample and library quality. We then pooled the libraries at an equimolar ratio and sequenced the libraries on one lane of 2 × 150 S4 Illumina NovaSeq 6000 run at Globe Institute Sequencing Core. We increased the sequencing effort for seven of the historical samples to obtain deeper coverage with an extra run in a 2 × 150 S4 Illumina NovaSeq 6000 at Novogene UK. Historical genomes were sequenced to an average of 4.9× (ranging from 2.9× to 8.4×) (Table S2). Historical samples showed deamination patterns (< 10%) typically observed in ancient DNA samples (Figure S3).

### 2.3 FASTQ Trimming, Mapping and Quality Control

We trimmed reads and removed adapters from raw FASTQs with SeqPrep2 (https://github.com/jeizenga/SeqPrep2), retaining reads longer than 30 base pairs and with MQ > 20. We aligned the sequences to the maternal haplotype (GCF_028858705.1) using BWA 0.7.17 *mem* (default parameters) for modern samples and *aln* (‐l 1024 ‐n 0.03 ‐o 2) for historical samples (Li and Durbin 2009). We removed PCR duplicates with picard MarkDuplicates 2.27.5 (http://broadinstitute.github.io/picard/). We assessed DNA damage for historical samples using mapDamage v2 (Jónsson et al. 2013) and genome‐wide coverage with MosDepth v0.3.3 (Pedersen and Quinlan 2018). For specific analysis as detailed below, we downsampled BAM files to 4× or 10× coverage using samtools v1.10 (Danecek et al. 2021) with *samtools view ‐s*.

### 2.4 Genotype Calling and Filtering

We used snpAD v0.3.1.10 (Prüfer 2018) to call genotypes in samples with coverage higher than 5× in each autosomal chromosome. Next, we merged individual VCFs per autosome in a single file with bcftools v1.20 (Danecek et al. 2021) *merge* option. We kept only those variants with a read depth between 4 and 50 (included) and a minimum genotype quality of 30 with bcftools *filter*. Finally, we concatenated all VCF files with bcftools *concat*. All the analyses were restricted to the autosomes.

### 2.5 Demographic History

We used three approaches that apply different inference methods to estimate past demographic history in effective population size (*N*_e_) at different time‐scales with the modern samples. For the most recent demographic changes in the past 100 generations, we used linkage disequilibrium (LD) estimates with GONE (Santiago et al. 2020), with default parameters and a recombination rate of 3.42 cM/Mb (Cui et al. 2024). We randomised individuals and sites with a Jackknife strategy to obtain a distribution of *N*_e_ estimates. For estimates over the past 10,000 years, we used site frequency spectrum (SFS) data in StairwayPlot V2 (Liu and Fu 2015, 2020) with a generation time of 13 years (Gil de Weir 2006), a mutation rate of 1.45e‐8 (Zhang et al. 2014), and eight random breaks. We obtained the folded SFS with ANGSD (‐uniqueOnly 1 ‐remove_bads 1 ‐only_proper_pairs 1 ‐C 50 ‐baq 0 ‐minMapQ 30 ‐minQ 20 ‐doCounts 1 ‐GL 2 ‐doSaf 1; Korneliussen et al. 2014). Finally, to obtain the long‐term *N*_e_, we used Pairwise Sequentially Markovian Coalescent (PSMC) (Li and Durbin 2011) with the reference genome sample mapped to itself, as it has the highest coverage (66.54×). We ran *psmc* with the following parameters: ‐N30 ‐t5 ‐r5 ‐p ‘4+30*2+4+6+10’ following (Nadachowska‐Brzyska et al. 2015) and performed 100 independent bootstrap runs.

### 2.6 Relatedness

We used ANGSD (Korneliussen et al. 2014) to obtain the genotype likelihoods in binary format (‐doGlf3 ‐GL 2) separately in the modern and historical datasets with the following filters: ‐uniqueOnly 1 ‐remove_bads 1 [−noTrans 1 (only for historical samples)] ‐only_proper_pairs 1 ‐C 50 ‐skipTriallelic 1 ‐minMapQ 30 ‐doMajorMinor 1 ‐doMaf 1 ‐SNP_pval 1e‐6. With the resulting files, we ran NgsRelate v2 (Hanghøj et al. 2019) to obtain the theta kinship coefficient. Standard kinship distribution values were used to classify individuals as unrelated when their kinship coefficient is 0; third‐degree or higher relatives for values between 0 and 0.0625; second‐degree for values between 0.0625 and 0.1875; and first‐degree for values between 0.1875 and 0.375.

### 2.7 Sample Pedigree and Inbreeding Coefficients

We used the R package FamAgg (Rainer et al. 2016) to construct a pedigree of 1836 captive‐bred individuals from the Studbook (Liu 2018). We calculated inbreeding coefficients for all individuals using the function inbreeding from the ribd package (Vigeland 2020) and calculated the relatedness of sampled individuals using the kinship function of the FamAgg package, which calculates relatedness as the chance that a locus is identical between the two individuals. Here, in the absence of inbreeding, a parent and child would have a calculated kinship of 0.25, and a selfing cross equates to a kinship of 0.5. These kinship calculations account for inbreeding within the pedigree using a recursive algorithm (Lange 1997). However, the method assumes that all founders are unrelated and non‐inbred, which might not be true for the whooping crane population.

### 2.8 Heterozygosity

We estimated heterozygosity per sample using ANGSD (Korneliussen et al. 2014) and winSFS (Rasmussen et al. 2022). We first extracted a list of high‐quality sites by estimating genotype likelihoods with the following parameters: ‐uniqueOnly 1 ‐remove_bads 1 ‐only_proper_pairs 1 ‐rmTrans 1 ‐C 50 ‐minMapQ 20 ‐minQ 20 ‐setMinDepth 3 ‐setMaxDepth *$maxCov* [twice the coverage per sample] ‐doCounts 1 ‐doMajorMinor 1 ‐GL 2 ‐doGlf 2 ‐doMaf 2. Next, we ran ANGSD with the list of high‐quality sites to obtain their site allele frequencies (‐doSaf 1). Finally, we ran winSFS to obtain the site frequency spectrum (SFS) per sample.

For the comparison between historic and modern samples, we removed transitions to account for DNA damage; to test for the effect of different depths of coverage in our samples, we also calculated heterozygosity in BAM files downsampled to 4×.

For comparisons within modern samples, we estimated heterozygosity at all positions (including transitions) using BAM files downsampled to 10× with ANGSD using the following parameters: ‐uniqueOnly 1 ‐remove_bads 1 ‐only_proper_pairs 1 ‐noTrans 0 ‐C 50 ‐baq 0 ‐minMapQ 30 ‐minQ 20 ‐setMinDepth 3 ‐setMaxDepth 10 ‐doCounts 1 ‐GL 2 ‐doSaf 1. Then we employed winSFS as before.

Next, we evaluated how much genome‐wide genetic diversity the whooping crane has in comparison to other bird species with a range of conservation statuses (Table S3). For each species, we mapped raw sequencing reads to the reference genome with bwa 0.7.17 (Li and Durbin 2009) for Illumina sequenced genomes or pbmm2 v1.13.1 for genomes sequenced with Pacbio (https://github.com/PacificBiosciences/pbmm2) using default settings. Next, we estimated genome‐wide heterozygosity from the BAM files by first obtaining genotype likelihoods with ANGSD (Korneliussen et al. 2014) with the following parameters: ‐uniqueOnly 1 ‐remove_bads 1 ‐only_proper_pairs 1 ‐C 50 ‐baq 0 ‐minMapQ 30 ‐minQ 20 ‐setMinDepth $minDP ‐setMaxDepth $maxDP ‐doCounts 1 ‐nThreads 10 ‐GL 2 ‐doSaf 1. MinDP and MaxDP were set to ⅓ and two times the average coverage in each genome. Next, SFS was estimated with realSFS ‐fold 1. We included the heterozygosity estimate of the historic sample with the highest depth of coverage in the multi‐species comparison. To account for the different estimate obtained when retaining only transversion sites, we corrected the estimate using the transition/transversion ratio of 2.89, which we estimated from the modern samples.

### 2.9 Inbreeding With Runs of Homozygosity

We estimated Runs of Homozygosity (ROH) using ROHan (Renaud et al. 2019) on samples with coverage > 4×. For historical samples, we precalculated damage patterns by running the script estimateDamage.pl (on the 50 bp at 5′ and 3′ of reads) provided by the software. We used these estimates to identify ROH with a minimum size of 1 Mb on the autosomes with the following parameters: –size 1,000,000 ‐‐rohmu 2e‐5 ‐‐deam5p ${sample}.5p.prof ‐‐deam3p ${sample}.3p.prof ‐‐auto autosomes.txt.

For modern samples, we estimated ROH using the same parameters as above but without the damage pattern profile files. Given the differences in coverage between historical and modern samples, we also downsampled modern samples to 4× and re‐ran ROHan. When restricting the analysis to only modern samples, we downsampled them to 10× for comparison.

We estimated inbreeding age by converting ROH lengths (cM) to generation with the formula *G* = 100/(2 × cM) (Thompson 2013), using a 3.42 cM/Mb recombination rate (Cui et al. 2024) and a 13‐year generation time (Gil de Weir 2006).

### 2.10 ROHbin (Runs of Homozygosity Per Bin)

We used a custom approach, ROHbin (*Runs Of Homozygosity per bin*), to determine the private ROHs variation (as determined by low heterozygosity regions) for each wild and captive population. The method uses the heterozygosity values split into bins of 1 Mb (also 500 and 250 Kb) obtained from ROHan (see above) and leverages a statistical framework to estimate bins that contain unique variation in each of the tested groups. For each bin, ROHbin uses an empirical Bayes approach moderated *F*‐statistic with the R function *ebayes* from the limma package (Ritchie et al. 2015) in R v. 4.1 (R Core Team 2022) to determine statistical significance and effect size (as logFC) for each comparison. In short, it applies an empirical Bayes method to shrink the probe‐wise (e.g., 1 Mb bins) sample variances toward a common value and to augment the degrees of freedom for the individual variances (Smyth 2004). Once the variance has been shrunken, it calculates moderated *t*‐statistics leveraging this estimated parameter and returns a *p* value for each 1 Mb genome bin tested in our case, comparing heterozygosity between the captive and wild populations. We considered bins with a *p* value < 0.05 to be significantly different between groups and likely to contain private variation in the wild or captive population.

### 2.11 Population Effective Size

We estimated inbreeding effective size $\left(N_{e}^{f}\right)$ and variance effective size $\left(N_{e}^{V}\right)$ between the Founders_wildborn and Late_captive + Wild samples (separated by four generations, *t* = 4), based on equations in Waples (1991).

The inbreeding effective size $\left(N_{e}^{f}\right)$ reflects the rate at which individuals are becoming related, leading to loss of heterozygosity. $N_{e}^{f}$ was estimated using the increase in *F*_ROH_ per generations: $F=\frac{F_{t}-F_{0}}{t}$; $N_{e}^{f}=\frac{1}{2\mathrm{\Delta F}}$, where $F_{t}$ is the average *F*_ROH_ for Late_captive + Wild; $F_{0}$ is the Average *F*_ROH_ for Founders_wildborn; and *t* are generations separating both populations.

Variance effective size $\left(N_{e}^{V}\right)$ captures the rate of change in allele frequencies due to genetic drift and is calculated over generations using the harmonic mean population sizes. $N_{e}^{V}$ was estimated as: $N_{e}^{V}=\frac{t}{2\left(\hat{F}-\frac{1}{S}\right)}$; with $\hat{F}=\frac{{\left(p1-p2\right)}^{2}}{\bar{p}\left(1-\bar{p}\right)}$, where $\hat{F}$ is the standardised allele frequency change; *p*1 is the allele frequency in Founders_wildborn and *p*2 the allele frequency in Late_captive + Wild; $\bar{p}$ is the mean allele frequency per SNP; *S* is the harmonic mean of sample sizes (*x*2 in diploid) and *t* the number of generations between populations.

### 2.12 Genetic Load

#### 2.12.1 SnpEff

We polarised the discovered variants into ancestral and derived using four outgroups: the three closest ancestral nodes to the whooping crane (Figure S4A) from the B10K genome alignment of 363 avian species (Feng et al. 2020) and the reference genome of one sister species ( *Grus nigricollis* GCA_004360235) (Zhou et al. 2019). We obtained the ancestral nodes fasta sequence in the hal file (Feng et al. 2019) using hal2fasta (Hickey et al. 2013). We fragmented each fasta sequence into 150 bp long sequences using bedtools windowMaker v2.30.0 (Quinlan and Hall 2010) and subsequently mapped them to our whooping crane reference genome with bwa mem v0.7.17 (Li and Durbin 2009). We obtained variants using bcftools mpileup and bcftools call 1.15.1 (Danecek et al. 2021), and combined them to the whooping crane VCF with bcftools merge. We annotated putative deleterious variants with SnpEff (Cingolani et al. 2012) using the genome annotation for GCF_028858705.1 obtained with the NCBI Eukaryotic Genome Annotation Pipeline.

The modern samples genotypes were obtained from bam files downsampled to 10× coverage to account for coverage variation within modern samples and between historical and modern. We filtered the resulting VCF to remove fixed positions and keep variants with a genotyping rate of 40%. We also restricted the analysis to those variants with a 100% genotyping rate across the four outgroups. We considered only sites where all outgroups were homozygous reference (0/0), which we defined as the ancestral state. Any alternative allele present in the whooping crane was then considered derived, which SnpEff assumes to be the potentially deleterious variant. We required consensus across all outgroups to increase the reliability of polarisation by minimising the misidentification of ancestral alleles due to lineage‐specific substitutions or low‐confidence sites. With this filter, we retained 67.4% of the genotypes (38,096/56,517) and treated the derived allele as putatively deleterious and the reference as ancestral (Figure S4B).

From the annotated VCF we extracted those variants classified as high, moderate, and low impact. High‐impact variants are assumed to have a high (disruptive) impact in the protein, probably causing protein truncation, loss of function (LoF) or triggering nonsense‐mediated decay (i.e., stop codons, splice donor variant and splice acceptor, start codon lost, etc.). Moderate‐impact variants are non‐disruptive variants that might change protein effectiveness (i.e., missense variants). Low‐impact variants are mostly harmless or unlikely to change protein behaviour (i.e., synonymous variants). We considered only variants with a minimum read depth of 5 and a maximum depth of 20 (twice the average coverage for modern samples) and 16 (twice the highest coverage for historical samples). For the analysis including modern and historical samples, we performed two approaches to explore the effect of site filtering: (1) including all variants across historical and modern datasets, and (2) only the shared variants between modern and historical datasets.

To approximate the genome‐wide genetic load, we counted derived alleles relative to the outgroup ancestral alleles as putatively deleterious. We separated the total genetic load into heterozygous load and homozygous load by counting the number of derived alleles with low, moderate, and high predicted levels for homozygous alleles (multiplied by two) and heterozygous alleles. The homozygous counts are a proxy of the realised load (deleterious variants that express fitness effects); heterozygous counts are a proxy of the masked load (deleterious variants whose fitness effects remain largely hidden). Heterozygous variants can partially express their deleterious fitness effects (Bertorelle et al. 2022), but in the absence of dominance coefficients (*h*) estimates, we assumed that *h* ~0 for moderately and highly deleterious mutations (Charlesworth and Willis 2009).

To account for the effect of sequencing depth, we downsampled two modern samples to 5, 10 and 15× coverage (EB31_S103 and EB33_S105). Given that differences in depth of coverage between historical and modern samples can create biased allelic counts, we normalised the moderate and high‐impact allele counts (for both heterozygous and homozygous state) by the total low‐impact allele counts, effectively correcting for variant discovery power.

Next, we estimated the historical to modern frequency change of deleterious variants (low, moderate and high). For each category of variants, we estimated the observed allele frequency per site in each population (modern or historical). We first tested if the observed frequency in each category had a significant change over time with a binomial test. Then we calculated $R_{\mathrm{xy}}=F_{m}\times \left(1-F_{h}\right)/F_{h}\times \left(1-F_{m}\right)$ (Do et al. 2015), where $F_{m}$ represents the allele frequency in modern samples, and $F_{h}$ represents the allele frequency in historical samples. The *R*_*xy*_ values for both high and moderate categories were then normalised with the low *R*_*xy*_ value. We performed a jackknife analysis to estimate the variance around the *R*_*xy*_ values by excluding 1% sites each time, for 100 times, and calculated the corresponding *R*_*xy*_ values. We also computed the difference in allele frequencies of the deleterious variants between historical and modern (Δfrequency = *F*_modern_ − *F*_Historical_). Finally, we estimated the proportion of homozygous load found inside and outside runs of homozygosity (ROH) using the bedtoolsr R package (Patwardhan et al. 2019). The counts of homozygous load of each deleterious category were normalised by the proportion of the genome in ROH (*F*_ROH_) and the proportion of the genome outside ROH (1 − *F*_ROH_) for each sample.

#### 2.12.2 LoadLift

Next, we further assessed genetic load in the captive‐bred and wild populations using the LoadLift pipeline (Speak et al. 2024) to obtain CADD scores derived from model species (chicken). This analysis is complementary in different ways. First, contrary to SnpEff we retained CADD scores within evolutionary conserved regions of the genome (ultra conserved elements [UCEs]) as mutations in these regions are more likely to be deleterious in most genetic and environmental backgrounds (Snetkova et al. 2022). Also, since CADD scores are continuous, it allows us to estimate the genetic load components, that is, the realised load and masked load (Bertorelle et al. 2022). We limited the inference to the 0.72% portion of the genome contained in UCEs and their 2000 bp flanking regions using the Phyluce pipeline (Faircloth 2016). We retained sites within the UCE with CADD scores present in all 37 modern individuals, where the chicken reference genome and whooping crane sample data contained the same alleles and where sites were not fixed across all 37 samples (144/536,774 sites retained). CADD scores are PHRED scaled and therefore not additive, and hence, we converted the CADD scores into rank values. The 144 unique CADD scores were converted to their relative rank within all potential CADD scores in the whole chicken genome, chCADD scores are defined in Groß et al. (2020) as ${\text{chCADD}}_{i}=-10\log_{10}\frac{n_{i}}{N}$, where $n_{i}$ is the rank of the potential mutation and *N* is the 3,073,805,640 potential mutations across all chicken chromosomes. chCADD scores were converted to determine proportion (*n*_*i*_/*N*), for example, a chCADD score of 20 equates to a *n*_*i*_/*N* of 0.01. A scaled conversion was applied 1/*n*_*i*_ to act as a proxy for the selection coefficient. We must note, however, that this transformation using rank‐scores is only a crude proxy, and that it might not capture the complex and largely unknown distribution of selection coefficients. Hence, comparing the load components within and between individuals needs to be interpreted with caution.

The genetic load, realised load and masked load were calculated for every locus following the formulas in Bertorelle et al. (2022): $\text{Genetic load}=\sum_{i=1}^{L\left(\mathrm{hom}\right)}s_{i}+\sum_{j=1}^{L\left(\mathrm{het}\right)}0.5s_{j}$; $\text{Realized load}=\sum_{i=1}^{L\left(\mathrm{hom}\right)}s_{i}+\sum_{j=1}^{L\left(\mathrm{het}\right)}h_{j}s_{j}$; $\text{Masked load}=\sum_{j=1}^{L\left(\mathrm{het}\right)}\left(0.5-h_{j}\right)s_{j}$. The rank score of selection coefficient (*s*_*i*_) was inferred from their CADD score. The dominance coefficient (*h*_*j*_) was categorised in function of the CADD score; for variants with CADD < 10 we assumed *h* = 0.3, for 10 < CADD < 20 h = 0.15, for 20 < CADD < 30 h = 0.02, and CADD > 30 h = 0. We thus assumed there is a negative relationship between s and h and that more highly deleterious mutations are expected to be more recessive (Charlesworth and Willis 2009).

To investigate how the genetic load varies as a function of kinship (taken from the pedigree) and relatedness (estimated from genomic data), we analysed the resulting genetic load component values of offspring from hypothetical wild–wild, captive‐captive, and captive‐wild crosses, assuming Mendelian segregation ratios of the parental alleles (Speak et al. 2024). The bioinformatics and computational analyses were performed on Crop Diversity HPC, described by Percival‐Alwyn et al. (2024).

## 3 Results

### 3.1 Temporal Patterns of Genetic Diversity, Inbreeding and Genetic Load

We estimated the genome‐wide diversity of the 2012 captive‐born whooping crane reference genome individual (mapped short‐reads to 66.54× coverage). The obtained value of 0.0008 het × bp^−1^ is on the low end of the diversity range observed across 34 bird species from diverse phylogenetic lineages (Figure 1C). We obtained the same value with modern samples downsampled to 10× coverage, highlighting that this depth is sufficient to confidently estimate genome‐wide metrics. The genome‐wide diversity of whooping cranes before the historical population size minimum (sample MCZ‐220028 collected in 1889, with 6.99× coverage) is more than three times higher, 0.00296 het × bp^−1^ (Figure 1C). This represents a statistically significant 70% loss of heterozygosity through time (Figure 1D). These estimates are based on an average of ~608.9 million genotyped sites in historical samples (range: 390,002,370–769,540,477) and ~845.5 million sites in modern samples (range: 822,343,437–850,867,088) (Table S2). To avoid any potential bias related to differences in sequencing coverage (Figure S5), we estimated heterozygosity after downsampling the entire dataset to 4× and found the same pattern (Figure S6, average ~536.6 million sites in historical and 614.5 million sites in the modern samples, Table S2). The loss of heterozygosity is coupled with a marked increase of inbreeding over time as evidenced by the increased fraction of the genome in runs of homozygosity (*F*_ROH_) (Figure 1E). Historical individuals showed no evidence of inbreeding as on average only 0.5% of their genome was contained in ROH (0.08%–1.5%, with one outlier at 28.4%), while modern individuals consistently showed high *F*_ROH_ with an average of 9.6% of their genome in ROH (2.4%–17.9%), with 3.91% of the genome in ROHs longer than 10 Mb consistent with recent inbreeding (Figure 1E; Figure S7; Table S2).

**FIGURE 2:**
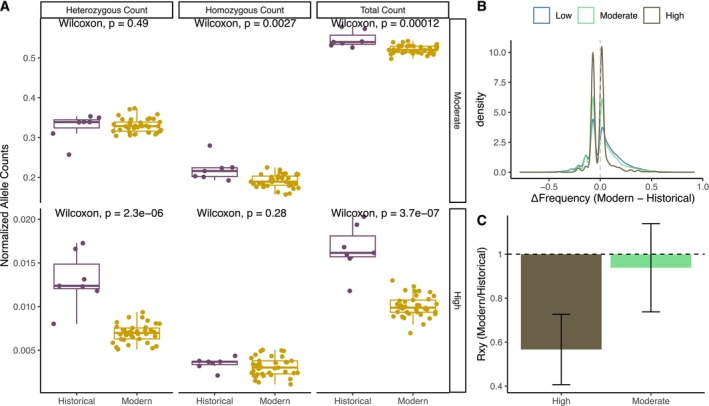
Temporal dynamics of genetic load. (A) Normalised derived allele count of deleterious variation (moderate and high impact) at heterozygous, homozygous and total load, estimated from ~40.2 million genotyped sites (Table S4). (B) Distribution of Δfrequency differences (Modern‐Historical) of deleterious variants (low, moderate and high). (C) *R*_*xy*_ ratio of derived alleles between modern and historical samples for the high and moderate impact deleterious variants (normalised by the low *R*_*xy*_). Normalised *R*_*xy*_ < 1 indicated relative frequency deficit of the corresponding category in the modern samples compared to the historical samples. Error bars represent the variance after jackknife resampling of variant sites.

The total count of deleterious alleles significantly decreased in modern populations, especially for the high impact variants (Figure 2A). In contrast, we observed no significant increase in homozygous counts over time, even though genetic drift is expected to convert heterozygous into homozygous derived alleles following a bottleneck. This may initially seem unexpected under classic drift theory, and we offer a technical and biological explanation.

Technically, comparisons of allele counts between historical and modern samples are sensitive to variant discovery biases due to differences in DNA quality, sequencing depth, and variant filtering. Including all confidently genotyped sites across both time points may underestimate rare and private variants in the historical samples, particularly those that were lost or fixed after the bottleneck. However, controlling for coverage effects, including normalising Moderate and High‐impact counts by Low‐impact variants (Figure S9), helps to mitigate most known biases. Moreover, when restricting the analysis to only variants that are shared between modern and historical datasets, we detect the expected pattern consistent with genetic drift and less efficient selection, and we observe a slight accumulation of load (Figure S8). This is expected because shared‐site filtering enriches for intermediate‐frequency variants, which are less likely to be lost or fixed. Consequently, such intermediate frequency variants are less biased in capturing allele frequency changes over time.

Biologically, the absence of increased homozygous load for highly deleterious variants is consistent with purifying selection acting against homozygous variants during or after the bottleneck. Strongly deleterious mutations are typically rare and recessive. Many of these variants are likely to have been lost randomly during the bottleneck, without ever becoming homozygous. Others will have been efficiently purged during the bottleneck, as homozygosity increased through inbreeding. Yet other variants would have increased in frequency by drift, but their rise would have been tempered by purifying selection. As a consequence of the combined effects of drift and purifying selection, we did not observe a significant increase in homozygous counts over time.

We further explored allele frequency dynamics to distinguish between purging and accumulation. Modern samples show a lower average frequency for high‐impact variants (Δfrequency = −0.0079) compared to moderate (Δfrequency = +0.0237) and low‐impact variants (Δfrequency = +0.0364). High‐impact variants also had a significantly smaller proportion of sites with positive frequency shifts (Figure 2B). These results indicate that selection has continued to act against strongly deleterious mutations, keeping their frequency low. In contrast, some moderate‐ and low‐impact variants have likely become effectively neutral s<12Ne due to reduced efficacy of selection, which allowed them to drift to higher frequency in the modern population.

To further test whether the purging of deleterious variants occurred during the extreme bottleneck, we calculated the ratio of derived alleles (*R*_*xy*_) between modern and historical samples for each impact category. We found a significant depletion of highly deleterious variants (Figure 2C). Overall, our results suggest that the genetic load dynamics are consistent with genetic drift and selection against high‐impact variants with s<12Ne. Most deleterious alleles were likely lost at random after the bottleneck, and some (mostly highly deleterious) were purged by purifying selection. At the same time, we observe a modest frequency rise of mildly deleterious variants, consistent with reduced selection efficacy following the population collapse.

### 3.2 Demographic History of Whooping Cranes

**FIGURE 3:**
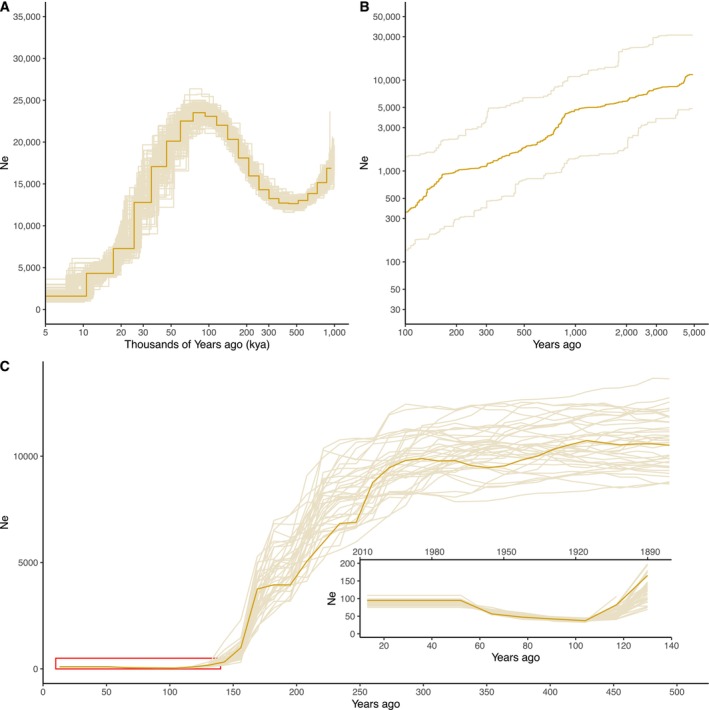
Demographic trajectory of the whooping crane through time. (A) PSMC analysis of the reference genome sample (66.54×). Thin lines represent 100 bootstrap replicates generated by resampling genomic segments with replacement. Analysis was performed assuming a mutation rate of *μ* = 1.45e‐8 (Zhang et al. 2014) mutations per site per generation and a generation time of 13 years (Gil de Weir 2006). (B) StairwayPlot of all modern samples. The plot shows the median effective population size over time, with shaded lines representing the 95% confidence interval defined by the 2.5th percentile and 97.5th percentile estimates. (C) GONE analysis using all modern samples in the last 500 years (or ~40 generations). Thin lines represent jackknife replicates, each generated by excluding one sample per run to assess variability in *N*_e_ estimates. The red square highlights the last 140 years as shown in the inserted plot at the bottom left.

Our PSMC reconstruction indicates that the whooping crane population has been relatively small for a long time (Figure 3A), with a harmonic mean *N*_e_ of 11,444 (between 5000 and 1 × 10^6^ years ago) and a maximum *N*_e_ of 23,515 (~80,000 years ago during the Late Pleistocene). After this point, our reconstruction from more recent time with StairwayPlot shows a steady decline that continues through the Holocene (Figure 3B) until recent times. A concordant signature picked up from reconstruction of the most recent past with GONE shows that the most recent bottleneck started around 300 years ago (mid‐17th century) and continued until 100 years ago (Figure 3C). At this point (eight generations ago, around 1916), we estimated the lowest *N*_e_ = 37.

### 3.3 Effect of Captive Management in Whooping Cranes

We integrated the pedigree information of the captive population and classified individuals according to their generation in captivity (Figure 1B; Table S2). The average pairwise kinship coefficient is 0.0095 (SD = 0.03), well below the third‐degree relatedness threshold (0.0625) (all estimates in Table S5). The estimated kinship within ‘Founders_wildborn’ individuals was relatively low, but increased in subsequent generations, within the ‘Early_captive’ cohort, only to be reduced again among the ‘Late_Captive’ (Figure S10), evidence that the captive population was likely founded with unrelated individuals and that captive breeding management has actively reduced relatedness.

**FIGURE 4:**
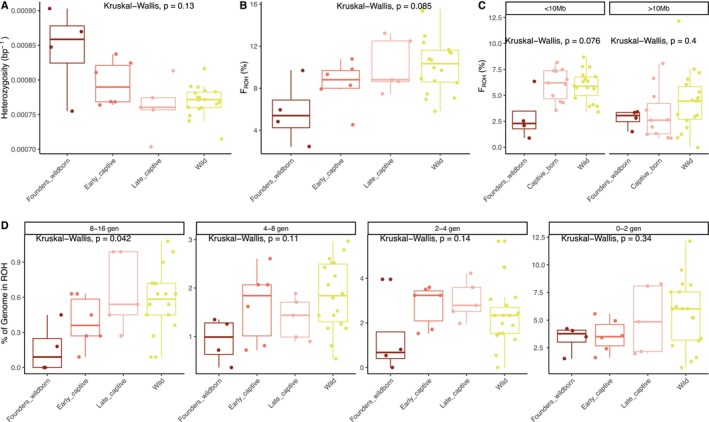
Genomic diversity and inbreeding in a captive time series and wild individuals. (A) Genome‐wide heterozygosity. (B) Inbreeding calculated as *F*_ROH_. (C) Inbreeding (*F*_ROH_) grouped by size: Smaller or larger than 10 Mb and merging early and late captive‐bred birds (Captive_born). (D) Inbreeding (*F*_ROH_) after stratifying ROHs by coalescence time (generations ago). In all plots, global statistical significance is calculated with a Kuskal–Wallis test.

We detected a non‐significant trend of loss of heterozygosity across captive generations (Figure 4A). Moreover, wild and ‘Late_Captive’ individuals, which represent a similar generation after the bottleneck, show similar levels of genome‐wide heterozygosity (Figure 4A). Inbreeding accumulated after the bottleneck in the captive breeding programme, but also in the wild unmanaged population (Founders_wildborn = 7.94%, Early_captive = 8.43%, Late_captive = 10.31% and Wild = 10.36%) (Figure 4B; Table S2; Figure S7). We used a dataset downsampled to 10× to avoid potential biases due to differences in coverage (Figure S11); we detected no difference in *F*_ROH_ using all data available or downsampling to 10× (Figure S12).

The coalescence time of most ROHs is within the last four generations or 50 years (Figure S13). Interestingly, the observed accumulation of *F*_ROH_ since the bottleneck is driven by ROHs smaller than 10 Mb (Figure 4C; Figure S14), reflecting background inbreeding due to past demographic events. In line with this evidence, we only identify a statistically significant increase in the proportion of the genome in ROHs for the oldest generation bin (8–16 generations ago, approximately 100–200 years ago) (Figure 4D). On the other hand, long ROHs have not accumulated between the first and last generation of captivity (Figure 4C). This may indicate that while captive breeding practices avoid inbreeding, the accumulation of background inbreeding was unavoidable in the absence of genomic data and due to the small founding captive population size (*N* = 35).

To support this interpretation, we estimated inbreeding effective size $\left(N_{e}^{f}\right)$ and variance effective size $\left(N_{e}^{V}\right)$ (Waples 1991) between the founder (Founders_wildborn) and the contemporary population (Late_captive + Wild samples) which are separated by approximately four generations. The inbreeding effective size $\left(N_{e}^{f}\right)$ reflects the rate at which individuals are becoming related, leading to loss of heterozygosity, whereas the variance effective size $\left(N_{e}^{V}\right)$ captures the rate of change in allele frequencies due to genetic drift. Our results show $N_{e}^{f}=83$, while $N_{e}^{V}=267.9$, which reinforce our findings of background inbreeding as a consequence of the peak of inbreeding after the bottleneck. In contrast, once the population rebounded and recovered to large numbers, $N_{e}^{V}$ increased substantially, even over just a few generations. These data show that, even after demographic rescue, background inbreeding is a serious concern for populations founded by few individuals and bottlenecked populations.

We did not detect any changes for the genetic load across successive captive generations or in the wild population (Figure S15). We identified the accumulation of genetic load inside ROHs in each captive generation and in the wild (Figure S16), consistent with reduced efficiency of purifying selection in regions of extended homozygosity. Interestingly, such accumulation was smaller and not statistically significant for high‐impact variants in late_captive and wild populations (Figure S16). This phenomenon detected for the most deleterious variants (in comparison to Moderate and Low impact) in later generations compared to earlier generations (Founders_wildborn and Early_captive) could be explained by selection efficiently removing ROH with highly deleterious mutations, but not ROH with mildly deleterious variation after the bottleneck.

**FIGURE 5:**
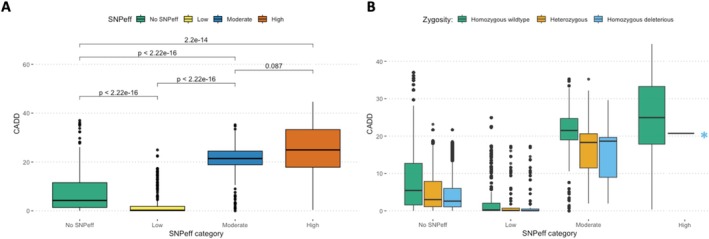
A comparison of lifted CADD scores and SnpEff categories across 37 whooping crane samples. (A) One‐to‐one comparison of SnpEff categories and CADD scores. (B) Zygosity compared across the different SnpEff categories, with individuals homozygous for the wildtype chicken reference allele (non‐scoring) (Green), heterozygous for the deleterious allele with a CADD score (Orange) and homozygous for the deleterious allele with a CADD score (Blue). A star (*) is used to denote that no sites categorised as highly deleterious by SnpEff were homozygous for the deleterious allele. The site counts of each category for both panels are the following: no SnpEff = 30,064, low = 258, moderate = 332 and high = 33.

The predicted deleterious scores from both SnpEff and CADD were consistent in ranking mutation severity. SnpEff‐classified deleterious mutations had higher average CADD scores than low‐impact mutations (moderate: 20.7, high: 23.5, low: 2.60; Wilcoxon rank sum, *p* < 2.2e‐16) (Figure 5A). For sites categorised by SnpEff as moderately deleterious, derived alleles (both heterozygous and homozygous) had significantly lower average CADD scores compared to homozygous wild‐type alleles across all modern samples (Wilcoxon rank sum, *p* < 2.2e‐16). Furthermore, no homozygous alleles were detected for mutations classified as highly deleterious with SnpEff and overlapping UCEs, aligning with the expected effects of strong purifying selection for highly deleterious variants in conserved regions (Figure 5B).

**FIGURE 6:**
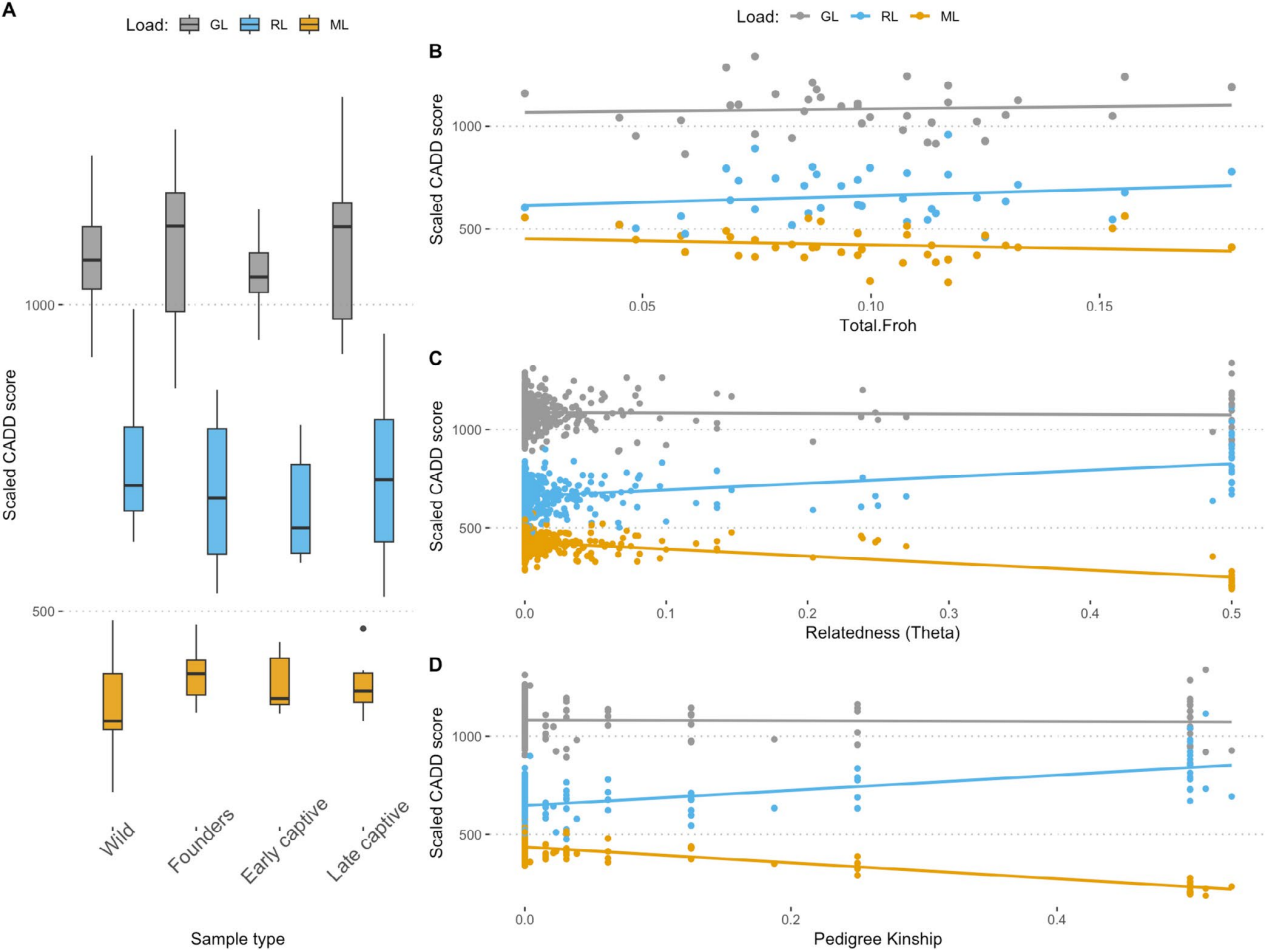
Genetic load components within ultra conserved elements (UCE) estimated from chicken CADD scores. (A) Individual genetic load was calculated for sites within the UCE the realised load (RL) (Orange), masked load (ML) (Blue) and the genetic load (GL) (Grey). (B) The GL, RL and ML of each of the 37 modern individuals as a function of their *F*_ROH_, (C) the GL, RL and ML of potential crosses compared to the genetic relatedness of the parents, and (D) the GL, RL and ML of potential offspring of crosses as a function of the relatedness of their parents based on the pedigree.

Converted CADD scores to genetic load estimates (see Section 2) revealed that the realised load exceeded the masked load in whooping cranes (Figure 6A), likely reflecting the effects of prolonged inbreeding and genetic drift from sustained small population sizes and serial bottlenecks. Inbreeding depression can be caused by a higher expression of genetic load in more inbred individuals; however, we detected no significant correlation between genetic load components and *F*_ROH_ (genetic load: *R* ^2^ = 0.0012, *p* = 0.8376; realised load: *R* ^2^ = 0.0111, *p* = 0.5342; masked load: *R* ^2^ = 0.0111, *p* = 0.5349). We proffer two biological explanations. First, ROHs contain mostly historical tracts already purged of highly deleterious mutations (Bosse and van Loon 2022). Second, due to the low level of masked load, inbreeding does not significantly elevate the realised load, which is consistent with our findings reported in Figure 2A (i.e., homozygous load does not increase over time). Finally, the lack of correlation could be explained by UCEs representing a very small proportion of the genome, in comparison of *F*_ROH_ that is genome‐wide.

We used converted CADD scores to predict the genetic load in offspring from hypothetical matings, assuming Mendelian segregation ratios, on 144 sites within the UCE (see Section 2). Results revealed that strategic pairings could slightly minimise expressed genetic load, as the predicted realised load and masked load were significantly affected by parental relatedness (realised load: *R* ^2^ = 0.2388, *p* = 2.272e‐08; masked load: *R* ^2^ = 0.6277, *p* < 2.2e‐16) (Figure 6C). Offspring from closely related pairs exhibited higher realised load, as masked load in the parents was converted into realised load in inbred offspring (Figure 6A). However, the rate of increase in realised load was modest compared to other heavily bottlenecked species, the pink pigeon (Speak et al. 2024), suggesting that most of the whooping crane's masked load has already been converted, rendering the species relatively robust to future inbreeding. Similar patterns emerged when using pedigree‐based relatedness values (genetic load: *R* ^2^ = 0.0035, *p* = 0.5872; realised load: *R* ^2^ = 0.2559, *p* = 6.75e‐07; masked load: *R* ^2^ = 0.6711, *p* < 2.2e‐16) (Figure 6D). These findings indicate that complete pedigree records in the captive program have effectively managed inbreeding and stabilised genetic load. Overall, while contemporary inbreeding may slightly increase realised load, this effect is likely to remain minor due to the current low masked load in the captive and wild populations.

### 3.4 Complementary Genetic Diversity Between Wild and Captive Populations

**FIGURE 7:**
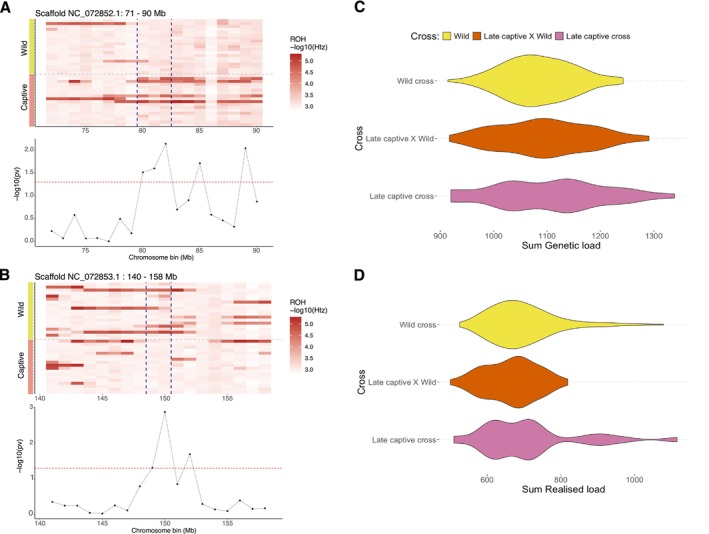
Complementarity of genetic diversity and genetic load between captive and wild populations. (A) Example of a region with significant enrichment of low heterozygosity in the captive population. ROHbin identifies regions private to each group, showing trends in low heterozygosity. (B) Example region with significant enrichment of ROH, private to wild populations. In panels (A, B), rows represent individuals, columns show 1 Mb genome bins, and colour intensity reflects heterozygosity (−log_10_ scale). (C) Distribution of genetic load across potential crossings. (D) Realised load from crosses between wild‐wild (yellow), wild‐captive (orange), and captive‐captive (pink) individuals.

Analysing 1 Mb genomic bins, we found that 5.1% of the wild genomes (~57 Mb) had significantly lower heterozygosity than the captive genomes (Figure 7A; Table S6), while 3.2% of the captive genomes (~35 Mb) showed lower diversity than the wild genomes (Figure 7B). These regions likely contain private genetic variation specific to each group. Excluding founders no longer contributing genetic diversity, the amount of private variation in the captive individuals (Late_captive) reduced to 1.16% (Table S6). These results align with the accumulation of *F*_ROH_ and ongoing diversity loss in captive generations (Figure 4B); though this analysis is limited by only six samples, reducing statistical power. All analyses yielded consistent results and patterns using smaller bin sizes of 500 or 250 Kb (Table S6).

Finally, we evaluated the genetic load in hypothetical optimal pairings to test whether captive‐wild crosses could reduce the realised load. The genetic load was similar for wild‐wild, captive‐captive, and captive‐wild crosses (Figure 7C). However, genomics‐informed selection of captive‐wild crosses could produce offspring with the lowest realised load by masking deleterious alleles (Figure 7D). There was no significant difference between average realised load of captive‐wild crosses and either wild‐wild or captive‐captive crosses (Kruskal–Wallis rank sum test, df = 2, *p* = 0.6738, *χ* ^2^ = 0.79). However, optimal captive‐wild crosses could reduce realised load by 44.44% compared to the worst captive‐captive pairings, and by 43.22% compared to the worst wild‐wild pairings, without significant increases in masked load (Figure S17). Similarly, the optimal captive‐wild cross could reduce realised load by 26.14% when compared to the mean of the five best crosses from all of the sampled individuals if selected based on pedigree kinship alone.

## 4 Discussion

The whooping crane faced extreme bottlenecks, nearly leading to extinction. Conservation actions, including habitat preservation, hunting restrictions, and breeding programmes, have since enabled population recovery to over 830 individuals across wild and captive populations, sparking debate on potential downlisting from Endangered to Threatened under the ESA and reassessment per the IUCN Red List (Caven et al. 2023).

In this study, we provide temporal genomic evidence of the severe consequences of the whooping crane's past population collapse. While we demonstrate the success of the captive breeding program in keeping recent inbreeding rates low, we also show that genomic erosion has not significantly improved (besides a small reduction in the most harmful genetic variants). We therefore caution against downlisting the species, arguing that its demographic recovery is not reflecting its continued poor genetic health. Contemporary whooping crane populations have lost approximately 70% of their ancestral genome‐wide diversity since 1893. Reduced heterozygosity could limit the population's ability to adapt to environmental changes (Femerling et al. 2023; Hoelzel et al. 2019; Hoffmann et al. 2017). While the most highly deleterious variants have likely been purged, remaining deleterious mutations still contribute to a prevalent realised load through recent time and across all populations. Together with Scandinavian grey wolf (Smeds and Ellegren 2023), Northern elephant seal (Hoffman et al. 2024), and possibly the Florida panther (Ochoa et al. 2022), the whooping crane belongs to a small and unenviable group of species with a realised load that exceeds their masked load. Increasing gene flow between captive and wild populations could help reduce the realised load and introduce novel genetic variation, potentially enhancing their long‐term viability.

### 4.1 Evolutionary Dynamics of Genomic Erosion Through Time

As previously described in other temporal genomic datasets from heavily bottlenecked species (e.g., Seychelles magpie‐robin—Cavill et al. 2024; Kākāpō—Dussex et al. 2021; Seychelles paradise flycatcher—Femerling et al. 2023; Crested ibis—Feng et al. 2019; Pink pigeon—Jackson et al. 2022; Cotton‐top tamarin—Rasmussen et al. 2023; Black rhino—Sánchez‐Barreiro et al. 2023; Eastern gorilla—van der Valk et al. 2019), whooping cranes show a strong (70%) reduction in genome‐wide diversity over time and marked inbreeding accumulation (Figure 1). Long‐term *N*_e_ estimates indicate the whooping crane declined over the past 1000 years. This trajectory aligns with the late‐Quaternary extinction (Bergman et al. 2023; Koch and Barnosky 2006; Svenning et al. 2024), which saw the extinction of two European crane species (*Grus primigenia* and *Grus melitensis*; Northcote 1984; Northcote and Mourer‐Chauviré 1988), a fate narrowly avoided by the whooping crane.

Still, the whooping crane population crashed even further in the last 300 years (Figure 3C), overlapping with European settlement of the Americas in areas where the whooping crane nested. Historical records show the population size dropped to around 16 individuals in the 1940s; our recent genomic *N*_e_ estimates are similar (*N*_e_ = 37 around 1916). The temporal analysis of genome‐wide heterozygosity loss offers greater confidence in *N*_e_ estimates. A loss of 70% since 1893 or 9.2 generations is equivalent to the level of genomic erosion expected in a population with *N*_e_ = 3.8, underscoring the fragile genetic health of the population. The *N*_e_ estimates at similar time points differ markedly across the three demographic reconstruction methods—likely due to their reliance on different data sources (Nadachowska‐Brzyska et al. 2022). Nevertheless, their trends are highly consistent. This allows us to confidently infer that: (1) the ancestral whooping crane population was never large, (2) it has been in steady decline for the last 100,000 years, and (3) the population was already small during the continuous bottlenecks in the mid‐1800s and mid‐1940s.

Our analyses of runs of homozygosity (ROH) and inbreeding effective size $\left(N_{e}^{f}\right)$ and variance effective size $\left(N_{e}^{V}\right)$ show the risk of relying solely on demographic data when making conservation assessments. We show that even if the population size recovers, the persistent effects of historical inbreeding (i.e., background inbreeding) can remain visible long after the bottleneck in closed populations without gene flow. The input of new variation through de novo mutations is a slow process. And even though recombination can reduce the maximum length of runs of homozygosity (ROH), their total combined length (and hence *F*_ROH_) does not go down without the introduction of new haplotypes. Hence, genomic erosion due to background inbreeding continues to be a problem after a bottleneck if the population remains isolated without gene flow.

The whooping crane's demographic history, characterised by long‐term small *N*_e_ and a recent abrupt collapse, provides important context for understanding the dynamics of genetic load over time. We observed no increase in the homozygous allele count for highly deleterious variants between historical and modern samples, despite a significant decrease in heterozygous counts. Although drift is expected to convert heterozygous into homozygous load, our findings suggest that following the bottleneck and demographic recovery, strong deleterious alleles exposed in homozygous form may have been purged before they could accumulate. Moreover, given that these mutations are typically rare and recessive, many may have been lost by chance before reaching homozygosity during the bottleneck, reducing the chance for homozygous load to increase. This pattern contrasts with the theoretical expectations of increased realised load under drift alone (figure 1a in Dussex et al. 2023), but despite potential technical factors (see Results), the observed pattern aligns with a model that accounts for load purging and accumulation. This would correspond to figure 1b in Dussex et al. (2023), but where the slope of the hypothetical purging line plotted against inbreeding coefficient is steeper than the slope of the masked load. Given the low masked load in the whooping crane, this explanation seems reasonable. Supporting this theoretical interpretation, we observed reduced allele counts, lack of frequency increase, and significant *R*_*xy*_ for high impact variants. In contrast, we detected a modest accumulation of mildly deleterious variants, as reflected in the positive frequency shifts and deleterious enrichment within ROH for moderate variants, and in the elevated ratio of realised to masked load in modern genomes based on CADD scores. This pattern mirrors what has been observed in other heavily bottlenecked species, such as the Alpine Ibex (Grossen et al. 2020), suggesting that purging of highly deleterious variants may have enhanced the species' resilience to inbreeding depression, but accumulation of mildly deleterious mutations could compromise fitness and should be monitored in ongoing conservation efforts in future generations due to ongoing inbreeding or genetic drift.

### 4.2 Captive Breeding Genomics

A common challenge for breeding programmes is that they are often established with a small founding stock of assumed unrelated individuals (Willoughby et al. 2017). The whooping crane captive population was founded with 35 individuals (Mirande et al. 1991). Here, we sampled six of these founders and found that pairwise kinship values among them were lower than expected for a third‐degree relationship. In the next generation in captivity, relatedness increased, probably due to a still very small captive population size. But in further generations this value went down, as the breeding programme avoided pairing closely related individuals using the rich pedigree information in this species.

Although the captive breeding program has avoided crosses between highly related individuals, loss of heterozygosity and accumulation of ROHs have continued across generations. This highlights the long‐term genetic impact of founder events, where small initial populations continue to affect diversity despite inbreeding management (Boakes et al. 2007). Notably, the accumulated inbreeding is mostly due to short ROHs, suggesting the captive program effectively prevents long ROHs from recent inbreeding, but can do little to avoid background inbreeding. These findings emphasise the importance of maintaining a well‐documented and complete pedigree, but also highlight that genomic data is crucial to supplement pedigrees, allowing for the accounting of cryptic relatedness and background inbreeding. Our analysis of in silico optimal mate pairings illustrates this by showing that reduced kinship crosses could help maintain a lower realised load.

Our CADD analysis assumes a range of dominance coefficients for estimating the load components within ultra conserved elements (UCEs), and thus is not directly comparable to the genome‐wide SnpEff estimates. It is worth noting that CADD scores are PHRED scaled and therefore not additive. As a result, the rank‐scores are only an approximation of the largely unknown distribution of selection coefficients. Also, the frequency, selection, and dominance coefficients of variants at UCEs may differ from those elsewhere in the genome. For example, an analysis of 29,938 polymorphisms within 2189 UCEs in the human genome showed that the polymorphism density within UCEs (one per 22.7 bp) was higher than the genomic average (one per 21.0 bp). However, the frequency of the derived allele was extremely low (less than 6% occur at a frequency of > 1%) (Habic et al. 2019). This underscores the need to better understand how in silico genetic load predictions translate into fitness effects in endangered species. Therefore, the comparison of the load components within and between individuals needs to be interpreted with caution.

### 4.3 One Plan Approach to In‐Situ and Ex‐Situ Management

The One Plan Approach aims to integrate in‐situ and ex‐situ conservation efforts by involving all stakeholders (Sauve et al. 2022; Speak et al. 2024). We identified a modest amount of private variation in captive and wild populations, suggesting that combining genetic pools could preserve some genetic diversity and reduce inbreeding. Our genomics‐informed management (Speak et al. 2024) shows that selecting optimal pairs could reduce realised genetic load by 20.78% compared to random mating, theoretically improving fitness and population viability. Optimising crosses may have only a modest effect, as the whooping crane's realised load exceeds its masked load, suggesting that most masked load has already been converted to realised load, providing some resilience against future inbreeding depression.

One method to achieve genetic exchange is through egg collection from wild breeding sites, as cranes typically raise only one chick from two eggs (Bergeson et al. 2001). Studies suggest collecting one egg does not impact nesting success (Boyce et al. 2005; Thompson et al. 2025), though some argue it may reduce recruitment in years with favourable breeding conditions (Cannon et al. 2001). With minimal impact, genetic material could be transferred by collecting one egg from a two‐egg nest in the remnant population, transporting it with an incubator, and rearing it in captivity for release into reintroduced populations (Kuyt 1996). The same positive effect could be achieved by supplementing the captive population. The Canadian Wildlife Service and partners have explored collecting a small number of eggs at the remnant wild population (AWBP) to bolster captive genetic diversity (Compass Resource Management Ltd 2023). Our results suggest these concerted in‐situ and ex‐situ actions could offer genetic benefits to the reintroduced and captive populations.

Translocation efforts must be carefully managed. While increasing genetic diversity is essential, gene flow from the remnant population could inadvertently introduce masked load, potentially leading to higher realised load after future inbreeding. Further studies with larger sample sizes and fitness data could help clarify the genetic variation across captive and wild populations. For instance, gene flow from wild populations could counteract adaptations to captivity that may have accumulated in the captive population (Frankham 2008). Additionally, ecological and behavioural factors are crucial, as reintroduced populations (EMP and LNMP) exhibit higher adult mortality and lower recruitment than the remnant wild population (AWBP) (Szyszkoski et al. 2025; LDWF 2022; Thompson et al. 2022; Wilson et al. 2016). Given the success and larger size of the AWBP, genetic introductions from the captive population may be unnecessary. However, AWBP genetic variation could benefit captive and reintroduced populations that are not yet self‐sustaining (LDWF 2022; Thompson et al. 2022). For any population management actions intended to improve population genetics and conservation, outcomes should be closely monitored to determine effectiveness (Gitzen et al. 2016).

### 4.4 Translating Genomic Insights Into Effective Conservation Management

Our genomic analysis underscores the lasting impact of severe bottlenecks on whooping cranes and highlights the importance of genomically informed captive breeding to maintain genetic health and resilience. Despite demographic recovery through in‐situ and ex‐situ conservation action, our results indicate persistent effects of reduced *N*_e_ and increased genomic erosion within the wild and captive populations. Given these findings, we advise against downlisting the whooping crane from Endangered to Threatened under the ESA and from Endangered to Vulnerable under the IUCN Red List. We instead recommend maintaining and expanding current conservation actions. Furthermore, our findings underscore the necessity of integrating genomic data into both in‐situ and ex‐situ conservation efforts.

Our study also highlights the importance of sampling and biobanking populations before population bottlenecks take hold, to establish a genetic baseline and contextualise genomic estimates for future predictions and conservation actions (Cavill et al. 2024; Díez‐del‐Molino et al. 2018; Femerling et al. 2023; Jensen et al. 2022). Genomic tools, as used here, offer a comprehensive framework for identifying inbreeding depression, genetic load, and adaptive potential, supporting unified conservation strategies to improve species survival (van Oosterhout et al. 2022; Segelbacher et al. 2022). By working with the International Crane Foundation and utilising these tools, we are taking critical steps toward data‐driven decisions that enhance genetic diversity, reduce inbreeding risks, and improve long‐term recovery outcomes for the whooping crane.

## Author Contributions

Design of the study: H.E.M. Sample collection by: H.E.M., B.K.H. Resources by: H.E.M., M.T.P.G., B.S., E.D.J. Laboratory work by: M.C.‐J., G.F., J.B. Data analysis: C.F., H.E.M., S.A.S., G.F., C.P., X.W., J.A.R., B.M., J.C., Y.S., L.A. Data interpretation: C.F., H.E.M., A.J.C., S.A.S., C.O., J.A.R., O.F., E.D.J. Writing: C.F., H.E.M., A.J.C., S.A.S., C.O. Editing and final approval of the manuscript: All authors.

## Conflicts of Interest

The authors declare no conflicts of interest.

## Supporting information


**Data S1:** mec70088‐sup‐0001‐DataS1.zip.

## Data Availability

All raw data generated in this study has been uploaded to the European Nucleotide Archive (ENA; Accession no. PRJEB80530). The metadata of these samples can be found in Table S2. Code for data analysis can be accessed through GitHub: https://github.com/claudefa/WhoopingCrane_genomics, https://github.com/pollicipes/ROHbin and https://github.com/saspeak/LoadLift.
