# Supplementary material for: Persistent Genomic Erosion in Whooping Cranes Despite Demographic Recovery
Source: Mol Ecol. 2025 Aug 26;34(23):e70088. doi: 10.1111/mec.70088 (PMC12684310; doi:10.1111/mec.70088)
Supplement: Supplementary file 1 — Data S1: mec70088‐sup‐0001‐DataS1.zip. [file MEC-34-e70088-s001.zip › mec70088-sup-0001-TableS1-S6-FigureS1-S165@Fontsere_etal_whooping_crane_Supp_figures_R1.docx]

**Supplementary Information for:**

**Persistent genomic erosion in whooping cranes despite demographic recovery**

Claudia Fontsere, Samuel A. Speak, Andrew J. Caven, Juan Antonio Rodríguez, Xuejing Wang, Carolina Pacheco, Molly Cassatt-Johnstone, Georgette Femerling, Brigid Maloney, Jennifer Balacco, Joanna Collins, Ying Sims, Linelle Abueg, Olivier Fedrigo, Erich D. Jarvis, Barry K. Hartup, Beth Shapiro, M. Thomas P. Gilbert, Cock van Oosterhout, Hernán E. Morales

**Table of Contents:**

[Supplementary Tables 1](#_heading=h.c0387pugicg7)

[Table S1 2](#_heading=h.56tpaskoo5ob)

[Table S2 2](#_heading=h.wikkefshvhfe)

[Table S3 2](#_heading=h.1wrf4gs23nhj)

[Table S4 2](#_heading=h.pl1prtk1osbw)

[Table S5 2](#_heading=h.9rtrp99vyn88)

[Table S6 2](#_heading=h.s50e7lpguanr)

[Supplementary Figures 3](#_heading=h.cf8coasgr16v)

[Figure S1 3](#_heading=h.kdinnrx4na62)

[Figure S2 4](#_heading=h.w1i5fq4845x9)

[Figure S3 4](#_heading=h.mj27sqrze1lg)

[Figure S4 5](#_heading=h.g1mkbnxsaggc)

[Figure S5 6](#_heading=h.nemu7ln1fwce)

[Figure S6 6](#_heading=h.7znb94tymd86)

[Figure S7 7](#_heading=h.wv0zk5arihdf)

[Figure S8 7](#_heading=h.vfptpz47ee6w)

[Figure S9 8](#_heading=h.3deyt7na9ykl)

[Figure S10 8](#_heading=h.wajuowt650i2)

[Figure S11 9](#_heading=h.ebl0d2f9pj8r)

[Figure S12 9](#_heading=h.ur1t2wvs0kg5)

[Figure S13 10](#_heading=h.d8cezjjpco3i)

[Figure S14 10](#_heading=h.ofv6ctfyo0ue)

[Figure S15 11](#_heading=h.f7drbm2yc0r)

[Figure S16 12](#_heading=h.ky6vj4mukxkw)

[Figure S17 13](#_heading=h.o5cnn3tluv1w)

# Supplementary Tables

Supplementary tables can be found in the excel file: SupplementaryTables.xlsx.

## Table S1

Summary of the library construction and sequencing kits, sequencing technology and software used for assembling the genome (A). Assembly statistics for maternal (B) and paternal haplotypes (C). The maternal haplotype assembly is chosen as the reference.

## Table S2

Metadata of all sequenced whooping crane samples, including their origin, location, year of collection, coverage, studbook information, heterozygosity and inbreeding (F_ROH_).

## Table S3

Heterozygosity of a set of bird species with different IUCN red list status with their accession number and coverage.

## Table S4

Genetic load allele counts (by zygosity) per sample classified by impact categories: high, moderate and low, obtained with SnpEff. Also includes genotype missingness rate per sample. We have included all sites (including private variation in historical and modern samples) and only shared sites among both groups (historical and modern samples).

## Table S5

Relatedness estimated as theta (kinship coefficient) between each pair of captive and wild (modern) whooping cranes.

## Table S6

Summary of the private regions of the genome in ROH per group (Captive/Late_Captive vs Wild) using different bin sizes (250Kb, 500Kb and 1Mb). These regions likely contain private genetic variation for each group.

# Supplementary Figures

## Figure S1

**A** Maternal Haplotype


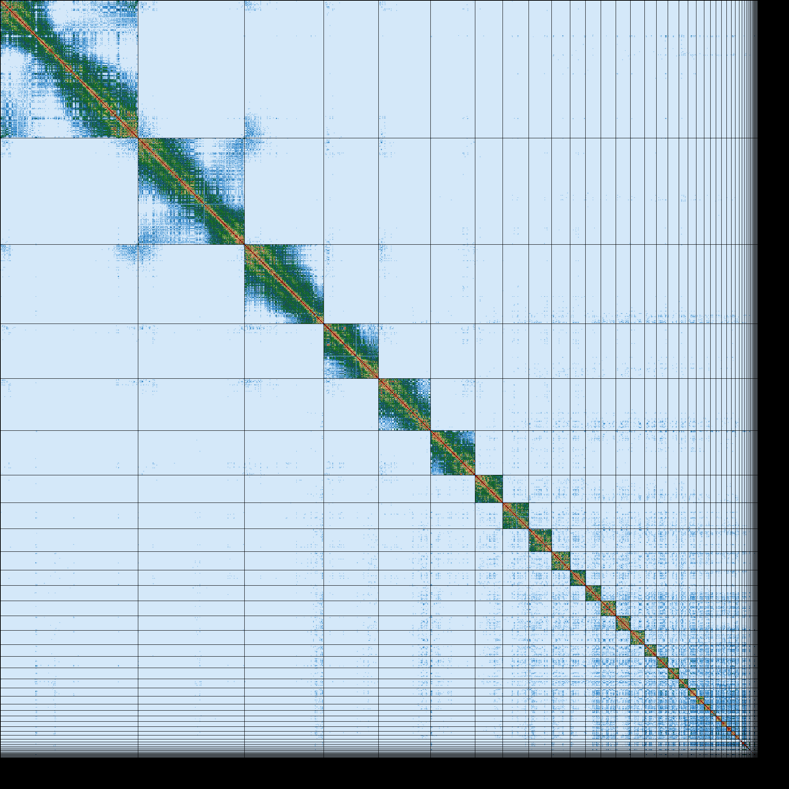


**B** Paternal Haplotype


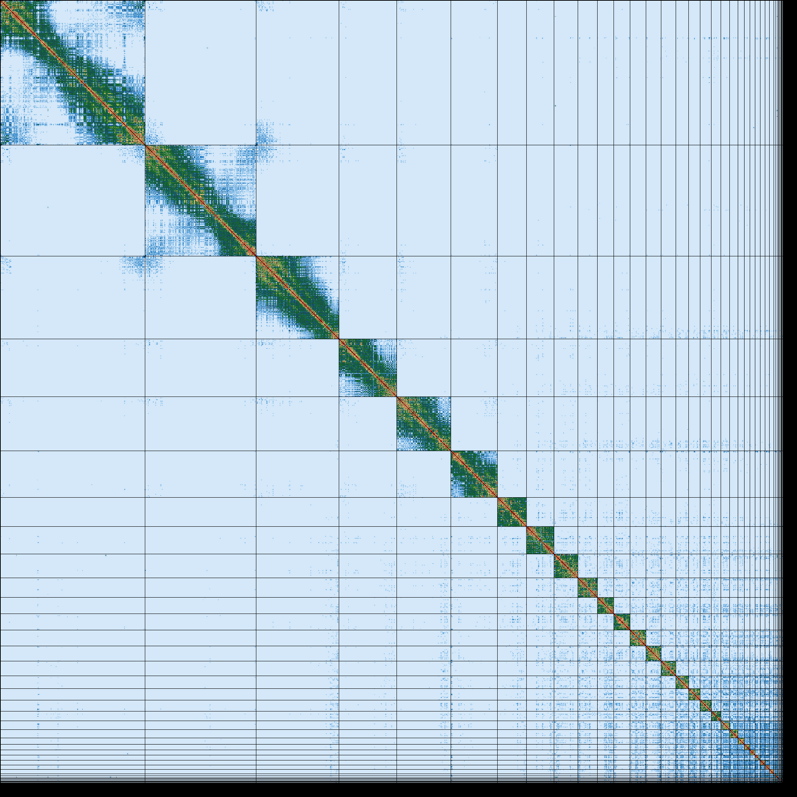


**Figure S1 | PretextMap showing HiC interactions for all scaffolds after manual curation.** **A)** Maternal haplotype and **B)** Paternal haplotype. No obvious missed joins or misassemblies are visible, and all chromosomes appear to be represented as chromosome-level scaffolds. A larger fraction of shrapnels is visible in the maternal haplotype compared to paternal.

## Figure S2

**A** **B**

Maternal Haplotype Paternal Haplotype


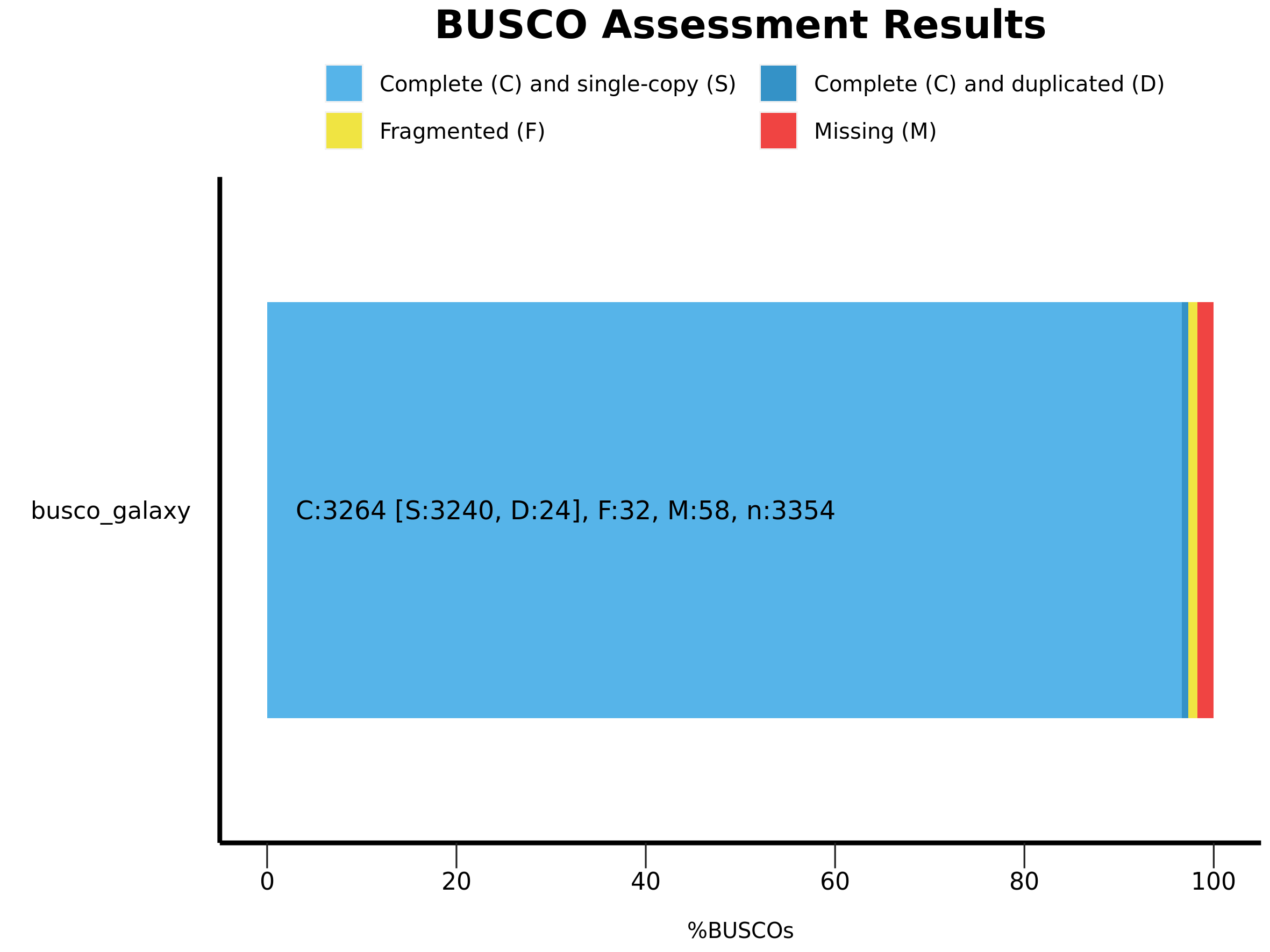


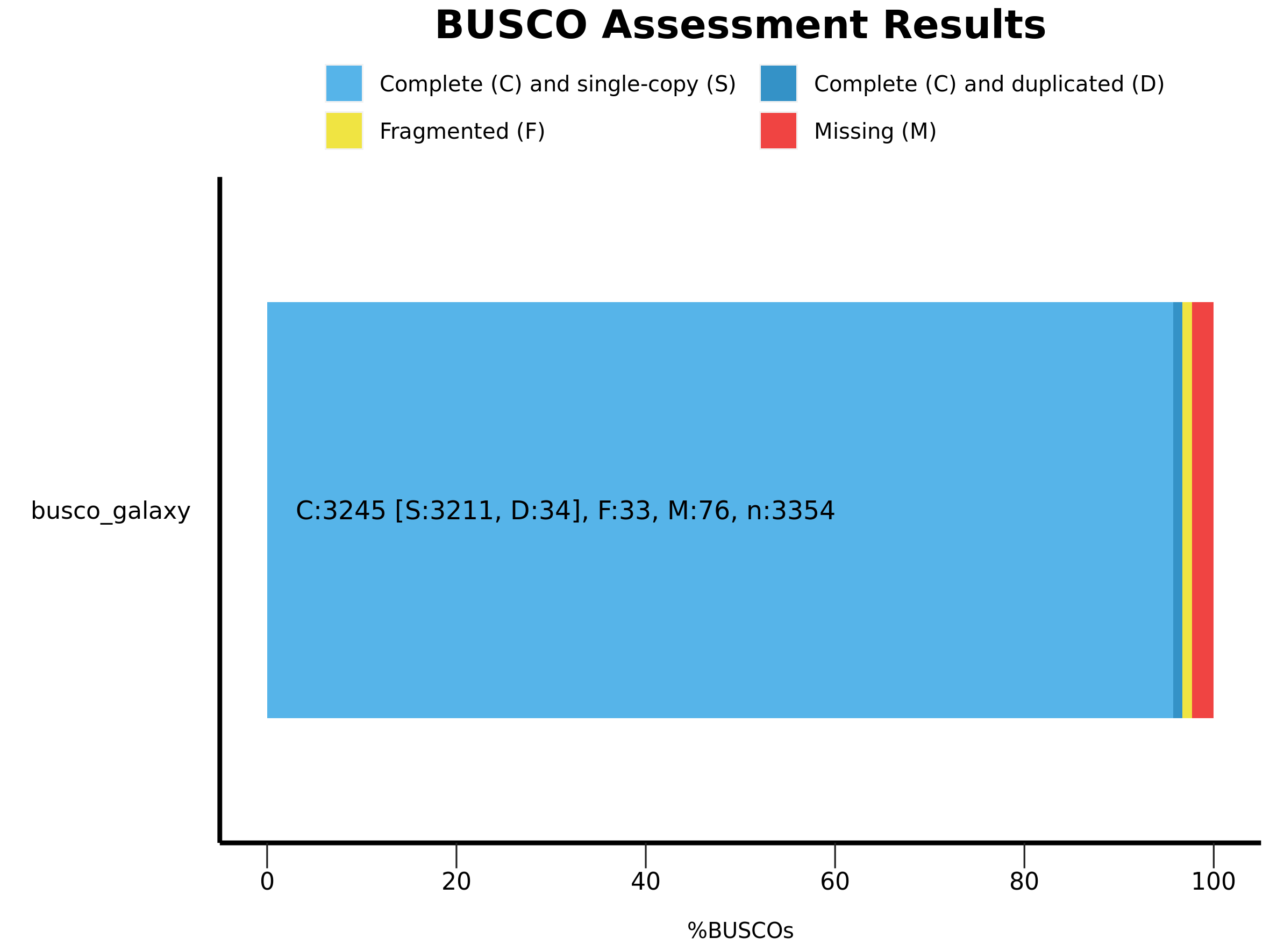


**Figure S2 | BUSCO summary report.** **A)** Maternal haplotype and **B)** Paternal haplotype. 1%, 2% or less genes appear duplicated or missing in both haplotypes, respectively. The maternal haplotype shows the smallest number of duplicated, missing, and fragmented genes, indicative of a slightly higher quality compared to paternal.

## Figure S3


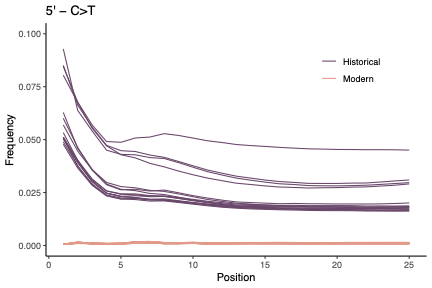


**Figure S3 | Damage patterns (C>T substitution) at 5’ of reads**, for historical and modern samples.

## Figure S4

**A**


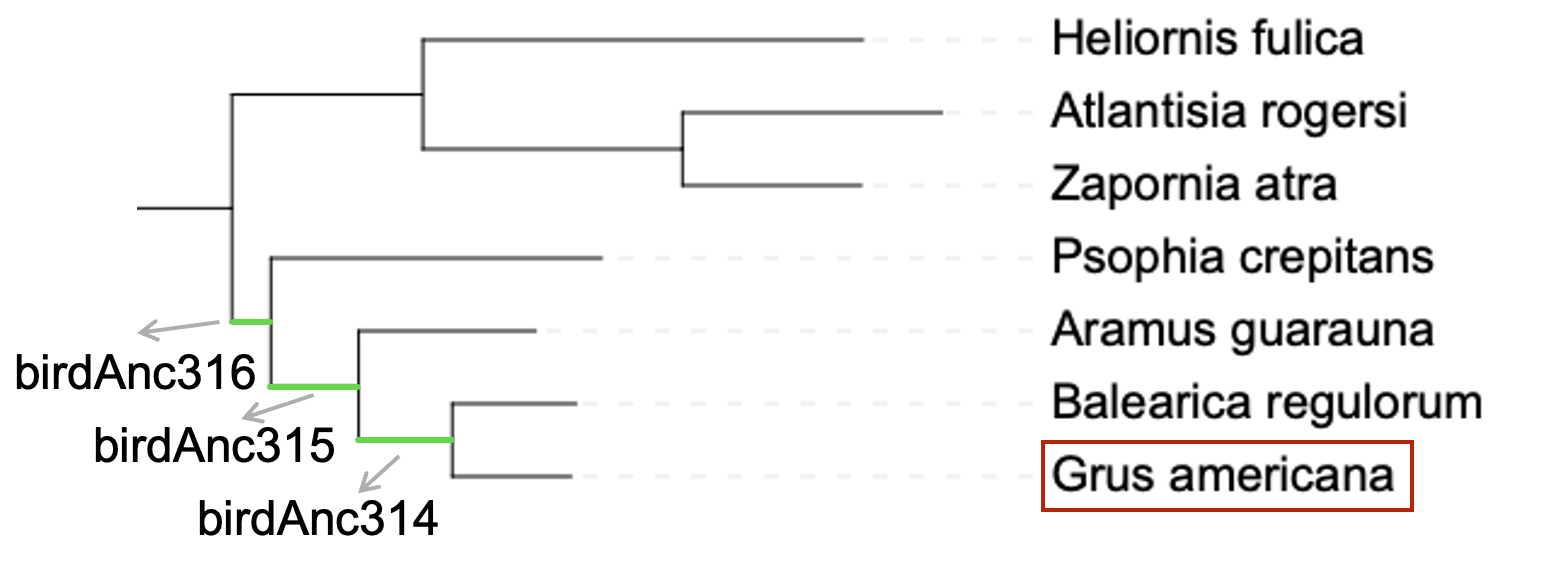


**B**


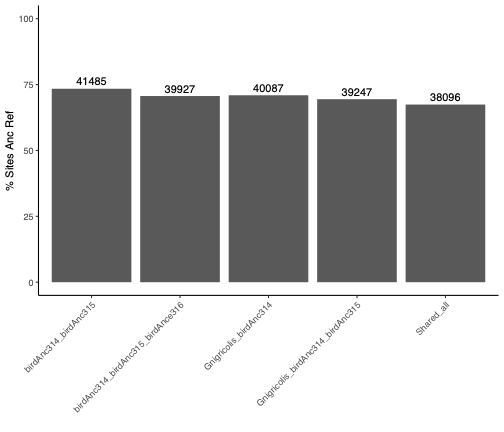


**Figure S4 | Ancestral allele polarization. A)** Phylogenetic positioning of the whooping crane (*Grus americana*) from [(Feng et al., 2020)](https://www.zotero.org/google-docs/?KpvKiq) to highlight the selected nodes for the polarization of ancestral and derived alleles in the whooping crane. **B) Percentage of reference sites polarized as ancestral using different combinations of ancestral nodes and the sister species (G. nigricollis). Total number of sites are found on top of each bar.**

## Figure S5


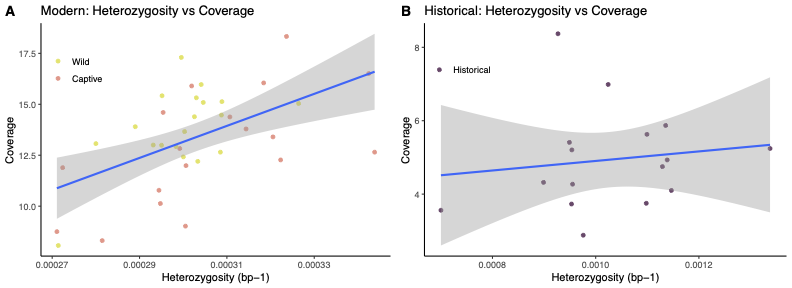


**Figure S5 | Correlation between depth of coverage and heterozygosity (only transversions)** in **A)** modern ( R-squared: 0.2913; p-value 0.0005657) and **B)** historical samples ( R-squared: 0.01873; p-value: 0.6132).

## Figure S6


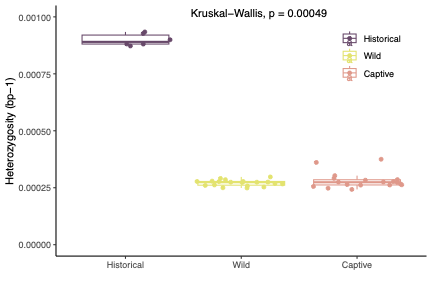


**Figure S6 | Genome-wide heterozygosity (only transversions) after downsampling bam files to an average coverage of 4x.** There is a statistically significant loss of genome-wide heterozygosity between historical, wild and captive whooping cranes (Kruskal-Wallis, p-value=0.00049)**.**

## Figure S7


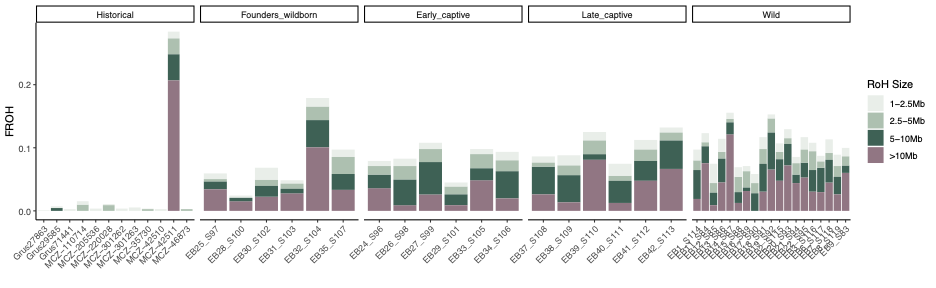


**Figure S7 | Proportion of the genome in Runs of Homozygosity (F_ROH_)** in samples with at least 4x coverage.

## Figure S8


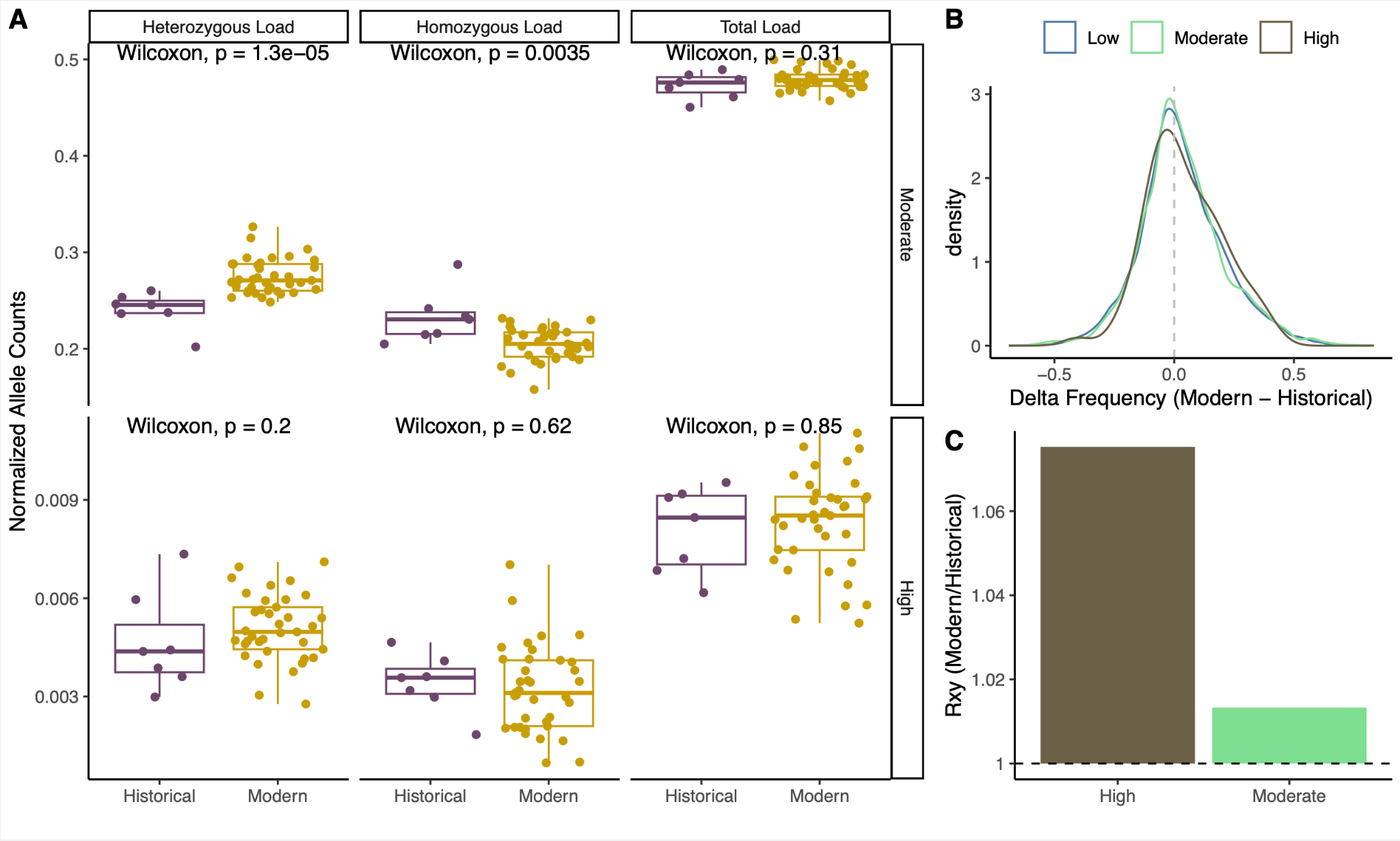


Figure S8 | Temporal dynamics of genetic load with only shared sites. A) Normalized derived allele count of deleterious variation (moderate and high impact) at heterozygous, homozygous and total load. B) Distribution of Δfrequency differences (Modern-Historical) of deleterious variants (low, moderate and high). C) Rxy ratio of derived alleles between modern and historical samples for the High and Moderate impact deleterious variants (normalized by the Low Rxy). Normalized Rxy<1 indicated relative frequency deficit of the corresponding category in the modern samples compared to the historical samples.

## Figure S9


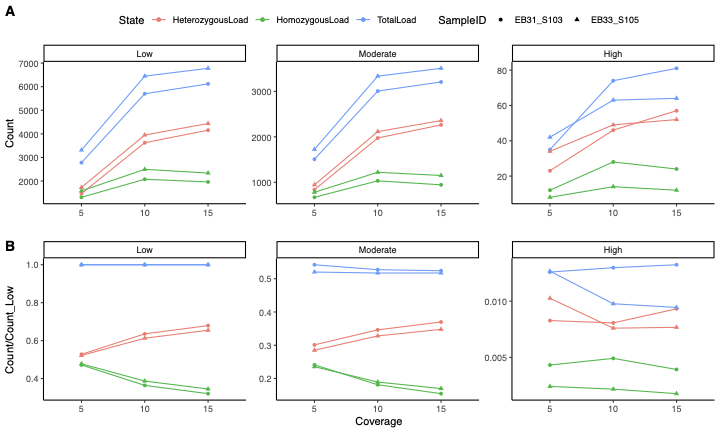


**Figure S9 | Effect of coverage in the detection of deleterious variants in two samples. A)** At 5x coverage the capacity to detect deleterious variants is impaired, especially for heterozygous load. Between 10x and 15x the difference is negligible. **B)** After normalization with total low allele count, the effect of low coverage is mitigated although there is still a tendency to overestimate homozygous load while underestimating heterozygous load.

## Figure S10


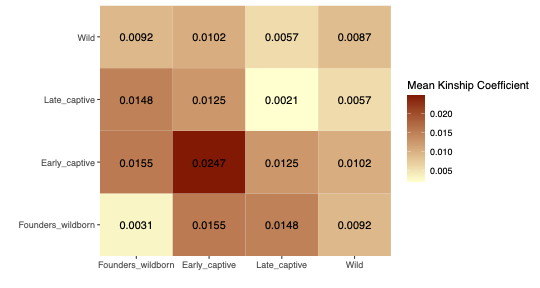


**Figure S10 | Average kinship coefficient (theta)** between and within captive and wild whooping cranes, estimated in a dataset of 3,485,136 SNPs including transitions.

## Figure S11


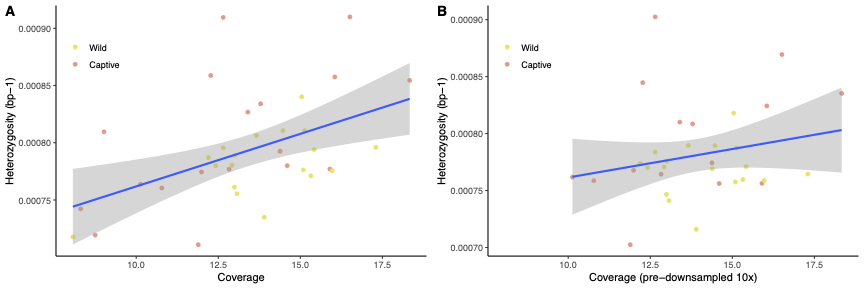


**Figure S11 | Correlation between depth of coverage and heterozygosity (all positions)** **for modern samples** in **A)** all dataset (adjusted R-squared: 0.4739, p-value: 5.715e-06) and **B)** after downsampling to 10x coverage and excluding samples with coverage <10x (adjusted R-squared: 0.02063, p-value: 0.2053).

## Figure S12


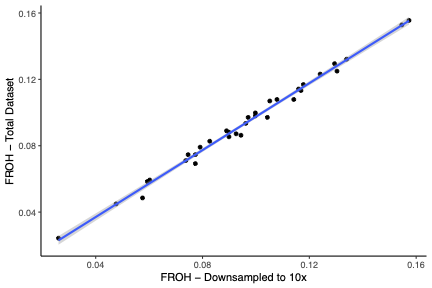


**Figure S12 | Comparison of F_ROH_** between the complete dataset and after downsampling to 10x (adjusted R-squared: 0.991, p-value: < 2.2e-16).

##

## Figure S13


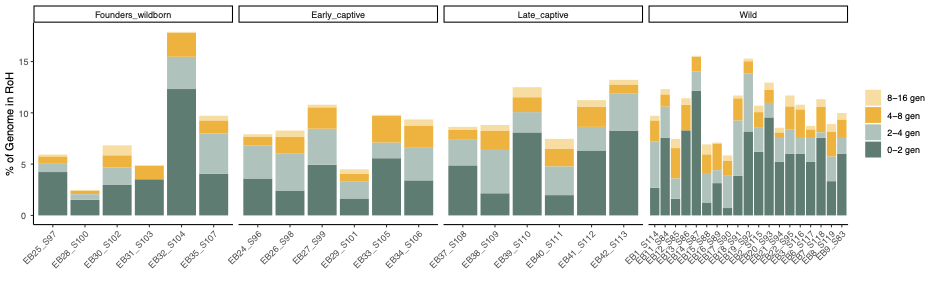


**Figure S13 | Percentage of the genome in Runs of Homozygosity (F_ROH_) stratified by their coalescence time** in modern samples with at least 4x coverage. We obtained the age of ROH in generations using a recombination rate of 3.42 cM/Mb and a generation time of 13 years.

## Figure S14


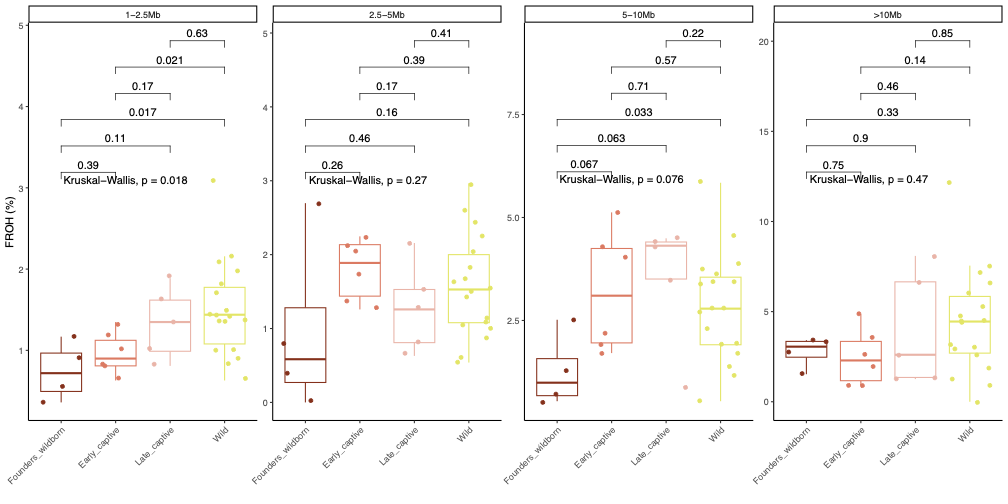


**Figure S14 | Accumulation of runs of homozygosity in different generations in captivity and in the wild.** F_ROH_ is segregated by ROH size: 1-2Mb, 2-5Mb, 5-10Mb and >10Mb. Statistical testing in pairwise comparisons are done with the Wilcoxon Rank Sum test, while the overall comparison with a Kruskal-Wallis test. This data only includes samples with at least 10x coverage.

## Figure S15


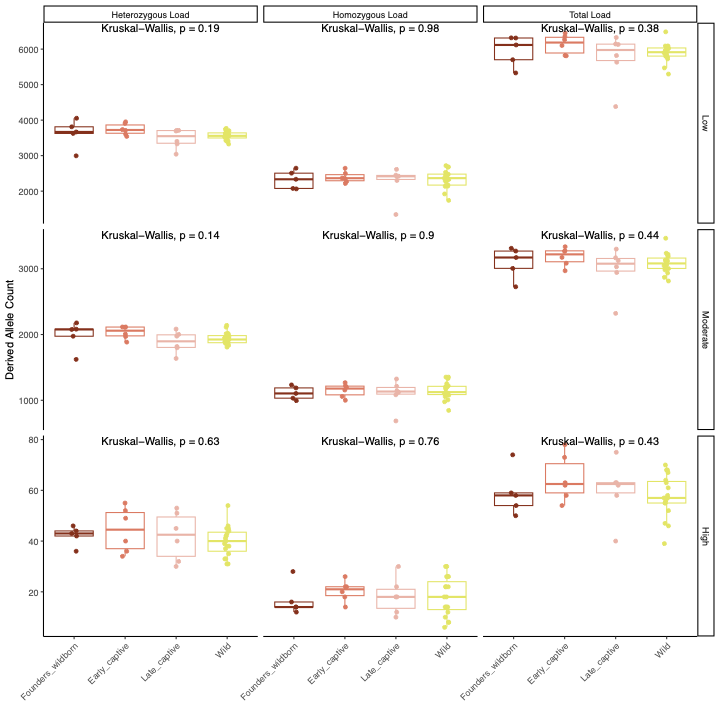


**Figure S15 | Genetic load in modern samples (downsampled to 10x)**. Genetic load is calculated by counting the derived allele in homozygosity, heterozygosity and total load (sum of both) for Low, Moderate and High impact variants. Statistical significance is obtained with a Kruskal-Wallis test for each category.

##

## Figure S16


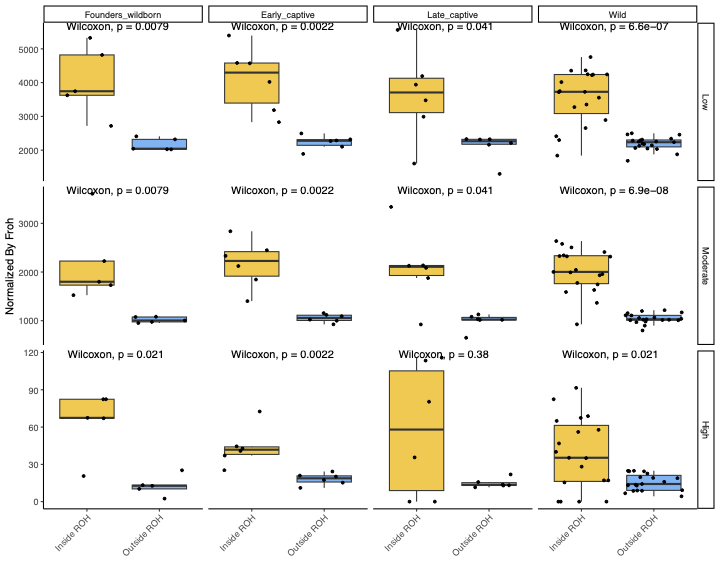


**Figure S16 | Proportion of homozygous load inside and outside ROH** as derived allele counts in homozygosity normalized by bases of the genome within ROH or outside ROH per sample In all plots, significance statistical tests are calculated with a Wilcoxon rank-sum test.

## Figure S17


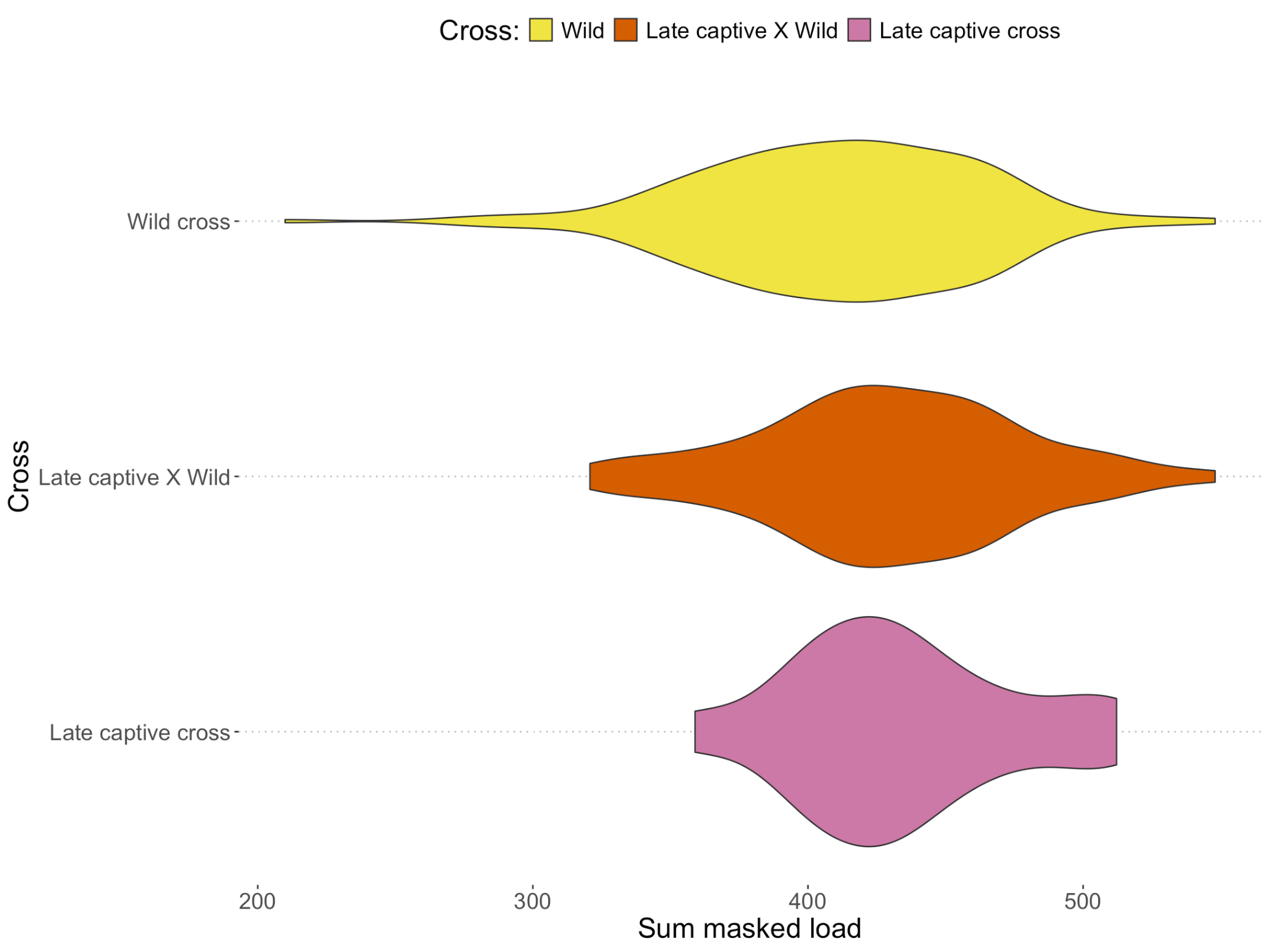


**Figure S17 | Masked load from potential** crosses between wild-wild (yellow), wild-captive (orange), and captive-captive (pink) individuals.
